# Niche Differentiation of Aerobic and Anaerobic Ammonia Oxidizers in a High Latitude Deep Oxygen Minimum Zone

**DOI:** 10.3389/fmicb.2019.02141

**Published:** 2019-09-13

**Authors:** Simone Muck, Daniele De Corte, Elisabeth L. Clifford, Barbara Bayer, Gerhard J. Herndl, Eva Sintes

**Affiliations:** ^1^Department of Limnology and Bio-Oceanography, Center of Functional Ecology, University of Vienna, Vienna, Austria; ^2^NIOZ, Department of Marine Microbiology and Biogeochemistry, Royal Netherlands Institute for Sea Research, Utrecht University, Den Burg, Netherlands; ^3^Research and Development Center for Marine Biosciences, Japan Agency for Marine-Earth Science and Technology (JAMSTEC), Yokosuka, Japan; ^4^Ecosystem Oceanography Group (GRECO), Instituto Español de Oceanografía, Centro Oceanográfico de Baleares, Palma, Spain

**Keywords:** ammonia oxidizers, denitrifiers, anammox, archaea, OMZ, Gulf of Alaska

## Abstract

To elucidate the potential for nitrification and denitrification processes in a high latitude deep oxygen minimum zone (OMZ) we determined the abundance and community composition of the main microbial players in the aerobic and anaerobic (anammox) ammonium oxidation and denitrification processes in the Gulf of Alaska throughout the water column. Within the dominant bacterial groups, Flavobacterales, Rhodobacterales, Actinomarinales, and SAR86 were more abundant in epipelagic waters and decreased with depth, whereas SAR11, SAR324, Marinimicrobia, and Thiomicrospirales increased their contribution to the bacterial community with depth. Nitrosopumilaceae also increased with depth and dominated the OMZ and bathypelagic archaeal communities. Euryarchaeota Marine Group II exhibited an opposite depth pattern to Nitrosopumilaceae, whereas Marine Group III and Woesearchaeota were more abundant in the bathypelagic realm. *Candidatus* Brocadia contributed 70–100% of the anammox bacterial community throughout the water column. Archaeal ammonia oxidizers (AOA) dominated the microbial community involved in the nitrogen cycle. Two AOA ecotypes, the high ammonia (HAC) and low ammonia (LAC)-AOA, characterized by distinct genes for aerobic ammonia oxidation (*amo*A) and for denitrification (*nir*K), exhibited a distinct distribution pattern related to depth and ammonia concentrations. HAC-AOA dominated in epipelagic (80.5 ± 28.3% of total AOA) oxygenated and ammonia-rich waters, and LAC-AOA dominated in the OMZ (90.9 ± 5.1%) and bathypelagic waters (85.5 ± 13.5%), characterized by lower oxygen and ammonia concentrations. Bacterial denitrifiers (3.7 ± 6.9 bacterial *nir*K gene mL^−1^) and anaerobic ammonia oxidizers (78 ± 322 anammox 16S rRNA genes L^−1^) were low in abundance under the oxygen conditions in the Gulf of Alaska throughout the water column. The widespread distribution of bacterial denitrifiers and anaerobic ammonia oxidizers in low abundances reveals a reservoir of genetic and metabolic potential ready to colonize the environment under the predicted increase of OMZs in the ocean. Taken together, our results reinforce the niche partitioning of archaeal ammonia oxidizers based on their distinct metabolic characteristics resulting in the dominance of LAC-AOA in a high latitude deep OMZ. Considering the different ecological roles and functions of the two archaeal ecotypes, the expansion of the zones dominated by the LAC-ecotype might have implications for the nitrogen cycle in the future ocean.

## Introduction

Microorganisms mediate most of the biogeochemical transformations in the global nitrogen (N) cycle (Kuypers et al., 2018). Over the last decade, fundamental pathways and key microbial players in the N cycle have been discovered (Kuypers et al., 2018). However, major uncertainties still exist on the extent and connection of nitrification and denitrification processes, especially in the open ocean (Ward and Jensen, 2014). The coupling of these processes, which affects the flow of N in the ecosystems, requires close interaction between nitrifying and denitrifying microorganisms, both spatially and/or temporally (Ward, 1996; Zehr and Ward, 2002). Oxygen minimum zones (OMZs) play an important role in the global ocean nitrogen cycle (Lam and Kuypers, 2011) providing an array of niches inhabited by metabolically diverse microorganisms (Bertagnolli and Stewart, 2018).

Nitrification is the two-step aerobic oxidation of ammonia (NH_3_) via nitrite (${\text{NO}}_{2}^{-}$) to nitrate (${\text{NO}}_{3}^{-}$), mediated by ammonia-oxidizing Archaea and Bacteria and nitrite-oxidizing Bacteria, respectively (Francis et al., 2005; Ward, 2011). Initially, the first stage of nitrification, the aerobic ammonia oxidation, was thought to be performed by ammonia-oxidizing bacteria (AOB) of the phylum Proteobacteria (Purkhold et al., 2000). However, the discovery of archaeal homologs of the bacterial genes encoding for ammonia monooxygenase (*amo*) in marine and terrestrial metagenomes (Venter et al., 2004; Treusch et al., 2005) and in an archaeal isolate (Könneke et al., 2005) led to the conclusion that members of the archaeal phylum Thaumarchaeota (formerly known as Marine Group I Crenarchaeota; Brochier-Armanet et al., 2008; Spang et al., 2010) are the main ammonia oxidizers in the ocean (Stahl and De La Torre, 2012). In contrast, AOB are present only in low abundances in the oceanic water column (Agogue et al., 2008). Recently, archaeal ammonia oxidizers (AOA) were differentiated into two vertically segregated clusters (Hallam et al., 2006; Beman et al., 2008), water cluster A (WCA) and cluster B (WCB). Later, two ecotypes were distinguished according to the environmental ammonia supply rates (Sintes et al., 2013). The high ammonia concentration (HAC) archaeal ammonia oxidizing ecotype, corresponding to WCA, is dominant in epipelagic and upper mesopelagic waters, especially at high latitudes, while the low ammonia concentration (LAC) (<10 nM) ecotype, corresponding to WCB, is dominant in the oligotrophic gyres and in meso- and bathypelagic waters (Sintes et al., 2013, 2016; Santoro et al., 2017). This general depth distribution of these subgroups might vary as described for the HAC ecotype or WCA, with a surface and a deep-water group (Sintes et al., 2016; Bertagnolli and Ulloa, 2017). Ammonia and nitrite oxidation have been observed in OMZs, even at low or non-detectable oxygen concentrations (Fussel et al., 2012; Kalvelage et al., 2013). Nitrite oxidation can exceed ammonia oxidation rates in these oxygen-deficient ecosystems (Fussel et al., 2012; Kalvelage et al., 2013), suggesting an uncoupling between these two processes. Bacterial nitrite oxidizers (NOB) are distributed throughout the OMZs and can account for a substantial proportion of the prokaryotic community in OMZs (Fussel et al., 2012).

The anaerobic ammonia oxidation (anammox) (Thamdrup and Dalsgaard, 2002), in which ${\text{NH}}_{4}^{+}$ is oxidized using ${\text{NO}}_{2}^{-}$ as electron acceptor and producing N_2_ gas (Jetten et al., 1998), has been suggested to be an important sink for fixed inorganic nitrogen in the ocean (Codispoti and Christensen, 1985). Globally, this process might be responsible for up to 50% of the N_2_ gas produced in the oceans (Dalsgaard et al., 2005). Several studies, based on phylogenetic analyses on the 16S rRNA gene (Brandes and Devol, 2002; Kuypers et al., 2005; Hamersley et al., 2007), indicate that this process is performed by members of the bacterial order Planctomycetales (Strous et al., 1999), represented by five *Candidatus* genera: *Candidatus* Brocadia (Strous et al., 1998), *Candidatus* Kuenenia (Schmid et al., 2000), *Candidatus* Scalindua (Schmid et al., 2003), *Candidatus* Anammoxoglobus (Kartal et al., 2007) and *Candidatus* Jettenia (Quan et al., 2008). Hydrazine-oxidoreductase (hzo) is the key enzyme of the anammox process (Shimamura et al., 2007), catalyzing the oxidation of hydrazine (N_2_H_4_) to N_2_. The hzo genes can be grouped into three clusters based on their phylogeny: hzo cluster 1, cluster 2a and 2b (Schmid et al., 2008). Bacteria of cluster 2 exhibit lower ammonia oxidation rates as compared to members of cluster 1 (Kartal et al., 2011). Up to now, few studies have focused on the abundance and community composition of anammox bacteria in the marine water column (Pitcher et al., 2011; Kong et al., 2013) and sediments (Dang et al., 2010, 2013; Shao et al., 2014) based on hzo genes (Schmid et al., 2008). Moreover, the dominance or co-existence of anammox and denitrification processes in oxygen-depleted environments is under debate (Dalsgaard et al., 2005; Bulow et al., 2010; Russ et al., 2014; Bristow et al., 2016). Overall, the balance between anammox and denitrification processes seems to be constrained by the flux and the C/N ratio of available organic matter (Babbin et al., 2014). The bioavailability of organic carbon and organic matter stoichiometry plays a key role in determining the relative contributions of anammox and denitrification to fixed nitrogen removal in the ocean (Dang and Chen, 2017).

Denitrification is an anaerobic respiratory process found in both autotrophic and heterotrophic prokaryotes (Ward et al., 2009), energetically less favorable than aerobic respiration (Deutsch et al., 2001) and predominantly taking place under anaerobic or low oxygen conditions. ${\text{NO}}_{3}^{-}$ and ${\text{NO}}_{2}^{-}$ are sequentially respired to NO and N_2_O, resulting in the production of N_2_. N_2_O gas has been reported to accumulate at the oxyclines in OMZs as a result of nitrification and denitrification and organic matter oxidation processes (Bange et al., 2001; Kock et al., 2016). Similarly, N_2_O concentrations in the Gulf of Alaska increase concurrently to the decrease in oxygen concentrations down to 600 m depth (Grundle et al., 2012). Several key enzymes are characteristic for denitrification processes and thus, can be used to assess the community composition and distribution of denitrifiers. Membrane-bound nitrate reductase (*nar*), encoded by the *nar*H and *nar*G genes, catalyzes the first step of dissimilatory nitrate reduction (Simon and Klotz, 2013). Nitrate reductase has been found in Firmicutes, Enterobacteria, Betaproteobacteria, and Gammaproteobacteria (Petri and Imhoff, 2000) in anoxic environments. The periplasmatic nitrate reductase, encoded by *nap*A, is expressed in the presence of nitrate (Imhoff, 2016) and is highly conserved in chemoautotrophic nitrate-reducing Epsilonproteobacteria (Vetriani et al., 2014). In general, denitrifying Bacteria harbor one of two types of nitrite reductases, either the cytochrome-containing enzyme encoded by *nir*S or the copper-containing enzyme encoded by *nir*K (Zumft, 1997; Petri and Imhoff, 2000). Previous studies have indicated a high diversity of Bacteria harboring *nir* genes within the Proteobacteria phylum (Braker et al., 1998; Huang et al., 2011). Interestingly, the *nir*K gene has also been found in bacterial and archaeal ammonia oxidizers (Casciotti and Ward, 2001; Treusch et al., 2005; Lund et al., 2012), and the *nir*S in anammox bacteria (Imhoff, 2016). Thus, it has been suggested that *nir*K and *amo* have co-evolved and that carrying both genes allow the cells to adapt to both aerobic and low oxygen environmental conditions (Treusch et al., 2005; Imhoff, 2016).

The aim of this study was to expand our knowledge on the coupling between aerobic and anaerobic ammonia oxidizing and denitrifying communities throughout the water column of the Gulf of Alaska (GoA). The GoA is characterized by the cyclonic Subarctic Alaskan Gyre, which comprises three major current systems (Stabeno et al., 2004). The southern boundary of the gyre is the West Wind Drift flowing eastward, which splits into the equatorward California Current and the poleward Alaska Current. The Alaska Current flows counterclockwise northward from ~48°N along the west coast of North America (Hickey and Royer, 2001) and becomes the narrow, fast flowing Alaskan Stream at the head of the Gulf, following the shelf-break south-westward. The Alaska Current is interspersed with frequent mesoscale anticyclonic eddies and meanders (Stabeno et al., 2004). Three types of eddies, which can persist for years, carry nutrient-rich warm waters offshore into regions with low ambient nutrient levels, and thus, can have a great impact on the chemistry and biology of the GoA ecosystem (Ladd et al., 2009). The basin is also well-known for its oxygen depleted deep waters (Hood and Zimmerman, 1986) and its seasonal deep OMZ (Paulmier and Ruiz-Pino, 2009).

We hypothesized that high latitude deep-ocean oxygen-limited areas might be characterized by bacterial and archaeal nitrifiers and denitrifiers different from the shallow tropical and subtropical OMZs due to distinctly different environmental conditions. To address the coupling or segregation of the different microbial nitrifiers and denitrifies, we used phylogenetic and functional marker genes to assess the distribution of two previously described ecotypes of AOA, *amo*A-HAC, and *amo*A-LAC (Sintes et al., 2013), and *nir*K harboring Archaea and Bacteria by q-PCR. Moreover, we characterized the community composition of Bacteria and Archaea, ammonia oxidizers, denitrifiers, and anammox in epi-, meso-, and bathypelagic waters. Additionally, we determined the environmental factors shaping the abundance and diversity of these communities. Our results support the previously suggested niche-separation of the two AOA ecotypes revealing a strong correlation between those ecotypes and two archaeal denitrification genes, indicating their co-occurrence within the same genome. Moreover, our results point to a larger contribution of the LAC-ecotype in deep ocean OMZs than in other, low latitude OMZs (Bertagnolli and Ulloa, 2017).

## Materials and Methods

### Study Area and Sampling

**Figure 1:**
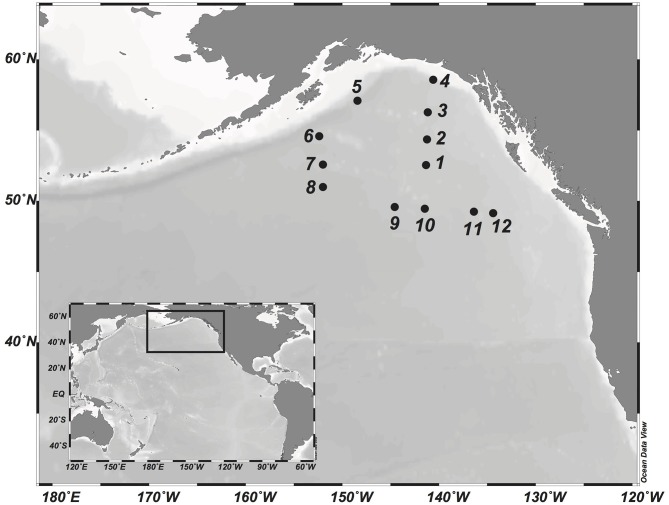
Map of the study area in the Gulf of Alaska. Sampling sites occupied during the DORC research cruise in 2013 are indicated by numbered dots.

Sampling was conducted in the Gulf of Alaska during the DORC (Deep Ocean Refractory Carbon) cruise on board R/V Melville in August 2013. Water samples were collected at 12 stations (Figure 1) at six depths for physico-chemical and biological parameters (bacterial abundance, DNA): the surface layer (5 m), the deep chlorophyll maximum (~50 m), the OMZ (located in the mesopelagic, ~1,000 m), the bathypelagic (at 2,000 and 3,000 m), and the bottom layer (max. depth 5,100 m). Water samples were collected with 12-L Niskin bottles mounted on a CTD (conductivity-temperature-depth) rosette sampler equipped with specific sensors for pressure (Paroscientific Digiquartz pressure transducer), temperature (SBE3plus), conductivity (SBE4C), and dissolved oxygen (SBE43). Salinity was determined on Guildline models 8400B and 8410 Portasal at the Marine Chemistry Laboratory at the University of Washington's School of Oceanography to calibrate the conductivity sensors (Hansell, 2016). Oxygen sensors were calibrated against water samples measured by Winkler titration (Langdon, 2010). Environmental parameters (depth, potential temperature, salinity, oxygen, AOU, nitrite, nitrate, and ammonia) are summarized in Table S1.

### Inorganic Nutrient Concentrations

Dissolved inorganic nutrient concentrations (${\text{NH}}_{4}^{+}$, ${\text{NO}}_{2}^{-}$, ${\text{NO}}_{3}^{-}$, ${\text{SiO}}_{4}^{4-}$, and ${\text{PO}}_{4}^{3-}$) were analyzed as described elsewhere (Hansell, 2016). The full dataset of measured inorganic nutrients can be downloaded from the Biological and Chemical Oceanography Data Management Office website (https://www.bco-dmo.org/dataset/527121).

### Abundance of Prokaryotes

Prokaryotic abundance was determined by flow cytometry following an established protocol (Brussaard, 2004). Water samples were fixed with glutaraldehyde (0.5% final concentration), flash-frozen in liquid nitrogen and stored at −80°C until further analysis. The samples were subsequently enumerated on a FACSAria II Cell sorter (Becton Dickinson) after staining with SybrGreen I based on their signature in green fluorescence vs. side scatter (Brussaard, 2004).

### DNA Extraction and PCR Amplification

Four to 9 L of water, depending on the expected prokaryotic abundance, were filtered onto 0.22 μm pore-size membrane filter (Millipore, GTTP), flash-frozen in liquid nitrogen and stored at −80°C until further processing in the lab. DNA extraction was performed using the UltraClean Soil DNA Isolation Kit (MoBio Laboratories) following the manufacturer's Advanced User's protocol. DNA extracts were stored at −80°C.

The primers used for amplification of phylogenetic and functional marker genes to generate q-PCR standards and for sequencing are listed in Tables S2, S3, respectively. The thermocycling conditions were chosen as previously described (Tables S2, S3) with slight modifications. The amplification reaction consisted of PicoMaxx Polymerase (2.5 U), 1x final concentration of PicoMaxx Reaction Buffer (Agilent Technologies), 1 μL DNA extract, primers (0.25 μM of *amo*A primers, 0.5 μM of archaeal *nir*K primers, and 0.4 μM of bacterial *nir*K primers, 0.5 μM of each primer set for sequencing purposes), 2 μg bovine serum albumin, 0.25 mM deoxyribonucleoside triphosphate equimolar solution mix, 5 mM MgCl_2_, made-up to 25 μL with PCR-grade water (Roche). The PCR products were checked by gel electrophoresis on a 2% agarose gel and visualized after staining with SYBR® Gold Nucleic Acid Gel Stain (ThermoFisher) for the right band size. The PCR product was purified using PCRExtract MiniKit (5-PRIME).

### Quantitative PCR

Q-PCR analysis was conducted on samples collected at the 12 stations and 6 depths per station (Figure 1). Q-PCR was used to quantify gene abundance of the bacterial *rec*A, the 16S rRNA of Archaea and the 16S rRNA of anammox Bacteria, as well as the abundance of the key functional genes of nitrification and denitrification pathways (archaeal *amo*A-HAC and *amo*A-LAC, archaeal *nir*K-a and *nir*K-b, and bacterial *nir*K) using specific primer sets (Table S2). The bacterial *nir*S gene was amplified with the primer set *nir*S-1F and *nir*S-6R (Braker et al., 1998), however, due to the ambiguous PCR product, the data have been excluded from further analysis. The recombinase A (*rec*A) was chosen as a proxy for bacterial abundance, as it is a single-copy gene in Bacteria (Lin et al., 2006). Standards for the different genes were prepared by amplifying a deep-sea sample or previously cloned PCR fragments (in the case of bacterial and archaeal *nir*K) with the specific primer sets as previously described (Sintes et al., 2013). For archaeal *amo*A-HAC, DNA from *Nitrosopumilus* cultures was used to prepare the standard (Sintes et al., 2013). Briefly, the amplified PCR products were checked on a 1% agarose gel stained with SYBR® Gold (Invitrogen) for the right size-band. Subsequently, the target PCR product was purified with the PCR Extract Mini Kit (5Prime). After the quantification of the purified products using a Nanodrop® spectrophotometer, the gene abundance was calculated from the concentration of the purified DNA and the corresponding fragment length. Triplicate 10-fold serial dilutions ranging from 10^0^ to 10^7^ specific gene copies were used to generate an internal quantification standard for each gene. The detection limit ranged between 1 gene copy μL^−1^ of extract (anammox 16S rRNA), 10 gene copies μL^−1^ (*rec*A, archaeal *nir*K-a, archaeal 16S rRNA, bacterial *nir*K), 100 gene copies μL^−1^ (HAC-*amo*A, LAC-*amo*A), and 1,000 gene copies μL^−1^ (archaeal *nir*K-b).

Q-PCR analyses were performed using the LightCycler 480 thermocycler (Roche) equipped with LightCycler 480 gene scanning software (version 1.5, Roche) as previously described (Sintes et al., 2016). The reaction mixtures for each gene contained 1 × LightCycler 480 SYBRGreen® I Master (Roche), primers (0.1 μM of *amo*A primers, 0.2 μM of archaeal *nir*K primers, and 0.15 μM of bacterial *nir*K primers), 1 μL of DNA extract (with nucleic acid concentration ranging between 1.8 and 44.6 ng μL^−1^) or 1μL of the corresponding standard dilution, and was made-up to 10 μL with PCR-grade water (Roche). Details on used primers and q-PCR thermocycling conditions are listed in Table S2. All standards, environmental samples, and negative controls were run in triplicate in 96-well q-PCR plates (Roche) with optical tape. The specificity of the q-PCR reaction was tested by gel electrophoresis (2% agarose) and by melting curve analysis (65–95°C) to identify unspecific PCR products. PCR efficiency was on average 78.1% for bacterial *rec*A, 83.9% for archaeal 16S rRNA, 92.6% for anammox 16S rRNA, 86.9% for archaeal *amo*A-HAC, 78.8% for archaeal *amo*A-LAC, 85.1% for archaeal *nir*K-a, 98.1% for archaeal *nir*K-b, and 74.4% for bacterial *nir*K. Gene abundance was normalized per mL of seawater considering the seawater volume filtered and the extracted DNA volume for each sample assuming 100% filtration and extraction efficiency.

Primer coverage of the HAC- and LAC-*amo*A was checked against the marine sequences from the most recent curated non-redundant database (Alves et al., 2018) using ARB (Ludwig et al., 2004). Allowing two mismatches, 35% of the *amo*A marine sequences were targeted by both primer sets, while ~35% of the sequences were only targeted by the HAC- primer set and 9% by the LAC- primer set, resulting in a total primer coverage of 79%.

The correlation between q-PCR quantification of the *rec*A gene abundance and prokaryotic cell enumeration assessed by flow cytometry indicated that *rec*A gene abundance was an appropriate proxy for prokaryotic abundance. A similar correlation between prokaryotic abundance and *rec*A gene abundance as found in this study was also found for the Atlantic (Steiner, 2013).

### Sequencing and Bioinformatic Analyses

Next generation sequencing was performed at IMGM Laboratories GmbH (Martinsried, Germany) on an Illumina MiSeq using v3 chemistry. Sequence libraries from three stations (Station 5, 10, and 11) and three depth layers (epi- meso- and bathypelagic) were constructed for the 16S rRNA gene of Bacteria, Archaea and anammox Bacteria, archaeal *amo*A, archaeal *nir*K-a and *nir*K-b, and bacterial *nir*K genes following the pipeline described previously (Sintes et al., 2016). These stations and depths were selected to cover distinct locations and contrasting environmental conditions (Figure 1, Figure S1). Primers and amplification conditions are listed in Table S3. All samples were barcoded using multiplex identifiers and sequenced together in one run.

The bioinformatics analysis of the 16S rRNA and functional genes followed the standard operating procedure pipeline (https://mothur.org/wiki/MiSeq_SOP) of Mothur (Schloss et al., 2009). The SILVA database (release 132) was used to identify the phylogenetic affiliation of the 16S rRNA gene. Briefly, the reads were quality checked using make.conting script implemented in Mothur. Sequences below 250 bp (below 200 for 16S rRNA of anammox bacteria) or larger than the corresponding fragment size (Tables S2, S3) were excluded. Sequences with ambiguities or more than 8 homopolymers were also excluded. The obtained sequences were further screened for chimeras using vsearch (Rognes et al., 2016) as implemented in the Mothur script chimera.vsearch with default settings. The sensitivity was decreased, however, for 16S rRNA of Bacteria (minimum score to report chimera increased to 5). Operational taxonomic units (OTUs) were defined as sequences with 97% similarity using the average linkage method cutoff implemented in Mothur. Phylogenetic diversity, Chao1, OTU richness, Shannon index of diversity, and the Simpson evenness index were calculated with Mothur using the subsampled OTU table to ensure equal number of OTUs for all samples for each of the different genes sequenced.

The sequence data of the 100 most abundant OTUs from each functional gene were aligned together with environmental and reference sequences from NCBI using MEGA-6 (Tamura et al., 2013). Phylogenetic trees of the functional genes were constructed in MEGA-6 (Tamura et al., 2013) using the Neighbor-Joining method (Saitou and Nei, 1987). Subsequently, the resulting trees were established using iTOL (Letunic and Bork, 2016). Obtained sequences were deposited at the sequence read archive at NCBI, accession number PRJNA507511.

### Statistical Analyses

Spearman's rank-order correlation analysis was performed using SPSS v20.0 (SPSS, Inc., Chicago, IL) to determine correlations between the community composition of the different phylogenetic and functional groups with environmental parameters (depth, potential temperature, salinity, apparent oxygen utilization, ammonia, nitrate, and nitrite concentration). Correlations were considered statistically significant at −0.5 > rs > 0.5 and *p* ≤ 0.05 for all analyses. Single correlation analysis was conducted in SPSS to test the relationship between prokaryotic abundance measured by flow cytometry and the *rec*A gene abundance assessed by q-PCR. Significant differences within phylogenetic and functional genes were assessed by the one-way ANOVA on ranks (Shapiro–Wilk normality test and the Mann–Whitney *U*-test) and *t-*test, both performed in Sigmaplot 12.0 (Systat Software, Chicago, IL, USA). The Canonical Correspondence Analysis (CCA) was performed using Past v.3.20 (Hammer et al., 2001) to test the relationship between the prokaryotic communities (bacterial and archaeal) and the environmental variables.

## Results

### Physico-Chemical Characteristics of the Water Column

Dissolved oxygen concentrations decreased with depth ranging between 225.10 and 298.70 μmol kg^−1^ in epipelagic waters to 11.80–23.60 μmol kg^−1^ at 1,000 m depth (Table S1). Consequently, the 1,000 m depth layer complies with the CRIO (CRIterion on O_2_) definition of an OMZ core (O_2_ < ~20 μM) (Paulmier and Ruiz-Pino, 2009). Below 1,000 m depth, oxygen concentrations increased toward the near bottom waters ranging between 111.00 and 152.40 μmol kg^−1^ corresponding to an apparent oxygen utilization (AOU) of 188.36 and 228.80 μmol kg^−1^. Nitrite concentrations were rather constant throughout the water column (≤0.89 μmol kg^−1^; Table S1). Nitrate concentrations generally increased with depth (0.23–46.47 μmol kg^−1^) reaching a maximum within the OMZ (38.88–46.47 μmol kg^−1^) and decreasing toward the deeper layers (≥33.35 μmol kg^−1^). Ammonia concentrations were typically highest in the epipelagic waters (≤3.03 μmol kg^−1^) and decreased with depth reaching minimum concentrations within the OMZ (≤0.01 μmol kg^−1^), and increasing again toward the bottom waters (≤3.19 μmol kg^−1^; Table S1).

### Prokaryotic Abundance

Total prokaryotic abundance determined by flow cytometry decreased with depth by one order of magnitude from an average of 4.7 × 10^5^ cells mL^−1^ in the epipelagic to 3.0 × 10^4^ cells mL^−1^ in bathypelagic waters (ANOVA on Ranks, *P* < 0.001). Total prokaryotic abundance was positively correlated with temperature, O_2_ concentration and nitrite concentration (*r* > 0.5, *p* = 0.000 for all three variables) and negatively correlated with depth, AOU, and salinity (*r* > −0.55, *p* = 0.000 for both variables).

**Figure 2:**
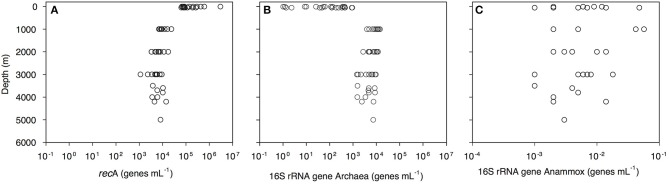
Depth profiles of **(A)** bacterial *rec*A, **(B)** archaeal 16S rRNA, and **(C)** anammox 16S rRNA gene abundance throughout the water column of the Gulf of Alaska.

Bacterial abundance, assessed as the *rec*A gene abundance determined by q-PCR, decreased with depth from 2.9 × 10^6^ genes mL^−1^ in the epipelagic to 1 × 10^4^ genes mL^−1^ in the bathypelagic waters (Figure 2A, Dataset S1). Archaeal 16S rRNA gene abundance increased from 1 gene mL^−1^ in the surface waters to 9.5 × 10^3^ genes mL^−1^ in the bathypelagic realm (Figure 2B). Highest archaeal 16S rRNA gene abundance was found in the OMZ (1.4 × 10^4^ genes mL^−1^; Figure 2B, Dataset S1). Archaeal 16S rRNA gene abundance was positively correlated with depth, salinity (both *r* > 0.5, *p* = 0.000) and AOU (*r* = 0.850, *p* = 0.000) and negatively correlated with temperature (*r* > −0.5, *p* = 0.000). Anammox bacterial 16S rRNA gene abundance was very low (highest abundance 57 × 10^−3^ genes mL^−1^) and without a clear depth distribution (Figure 2C). Prokaryotic abundance determined by flow cytometry was related to *rec*A gene abundance determined by q-PCR (y = 0.05x^1.15^, *r* = 0.80, *p* < 0.001).

### Abundance and Distribution of Archaeal Ammonia Oxidizers

**Figure 3:**
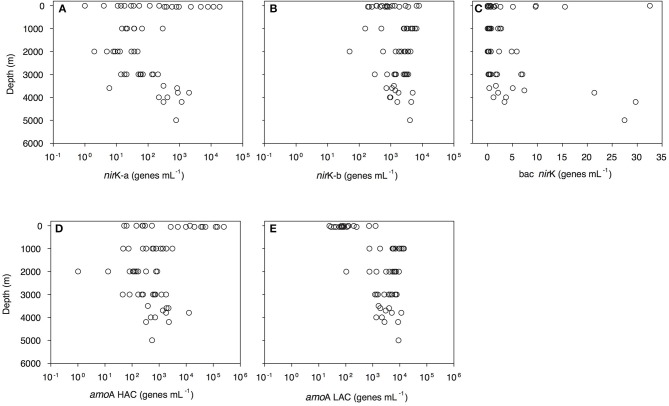
Depth profiles of functional gene abundances: archaeal *nir*K-a **(A)** and *nir*K-b **(B)** and bacterial nirK **(C)** archaeal HAC-*amo*A **(D)** and LAC-*amo*A **(E)** nitrification genes measured by q-PCR. *amo*A-HAC, “high-ammonia concentration” archaeal *amo*A; *amo*A-LAC, “low-ammonia concentration” archaeal *amo*A; *nir*K-a and *nir*K-b, archaeal nitrite reductase K type a and b; bac *nir*K, bacterial nitrite reductase K.

Archaeal *amo*A-HAC (high ammonia concentration *amo*A) gene abundance decreased from 0.5 × 10^2^-2.4 × 10^5^ genes mL^−1^ in epipelagic waters to 0.4–30.9 × 10^2^ genes mL^−1^ in the OMZ, and 1–12 × 10^3^ genes mL^−1^ in bathypelagic waters (Figure 3D, Dataset S1). In contrast, archaeal *amo*A-LAC (low ammonia concentration *amo*A) gene abundance increased from 0.3 to 12 × 10^2^ genes mL^−1^ in epipelagic waters up to 7.5–142.7 × 10^2^ genes mL^−1^ in the OMZ and 1.1–112.5 × 10^2^ genes mL^−1^ in bathypelagic waters (Figure 3E, Dataset S1). The ratio of *amo*A-HAC to *amo*A-LAC gene was ≤ 1 throughout the water column, with the exception of the 50 m depth layer (deep chlorophyll maximum, DCM), where it reached 10–2,837 (data not shown). The ratio of total *amo*A (i.e., HAC plus LAC) to archaeal 16S rRNA gene abundance showed a similar pattern, with most values close to 1 throughout the water column, but reaching a maximum value of 427 in the DCM (data not shown). The *amo*A-LAC gene abundance was significantly correlated to archaeal 16S rRNA gene abundance (y = 4.29x^0.86^, *r* = 0.88, *p* = 0.000). Furthermore, archaeal *amo*A-LAC gene abundance was positively correlated with depth, salinity, ${\text{PO}}_{4}^{3-}$ (*r* ≤ 0.53, *p* < 0.001 for all these variables), and AOU (*r* = 0.74, *p* < 0.001). Moreover, archaeal *amo*A-LAC abundance was negatively correlated with potential temperature (*r* = −0.51, *p* < 0.001) and ${\text{NO}}_{2}^{-}$ (*r* = −0.54, *p* < 0.001). The archaeal *amo*A-HAC: *amo*A-LAC ratio was positively correlated with O_2_ and ${\text{NO}}_{2}^{-}$ concentrations (*r* ≥ 0.5, *p* < 0.001) and negatively correlated with AOU, ${\text{PO}}_{4}^{3-}$ and ${\text{NO}}_{3}^{-}$ (−0.5 ≥ *r, p* < 0.001). No significant correlations were found between the abundance of archaeal *amo*A-HAC genes and the physico-chemical parameters (data not shown).

### Abundance and Distribution of nirK Containing Prokaryotes

Archaeal *nir*K gene abundance dominated over bacterial *nir*K genes (Figure 3, Dataset S1). Archaeal *nir*K-a gene abundance varied from 1 to 1 × 10^4^ genes mL^−1^ in epipelagic waters and from 1 to 294 genes mL^−1^ in the OMZ and 1–1 × 10^3^ genes mL^−1^ in bathypelagic waters (Figure 3A). Archaeal *nir*K-b gene abundance ranged between 1.9 and 78.7 × 10^2^ genes mL^−1^ in epipelagic waters, between 1.6 and 65.7 × 10^2^ genes mL^−1^ in the OMZ and between 0.5 and 49.5 × 10^2^ genes mL^−1^ in bathypelagic waters (Figure 3B). Overall, the abundance of *nir*K-a and *nir*K-b genes was significantly different (*P* < 0.001, *t*-test) throughout the water column. Bacterial *nir*K gene abundance was highest in surface and near-bottom layers, and lowest in the OMZ. Bacterial *nir*K gene abundance was ≤ 33 genes mL^−1^ in epipelagic waters, ≤ 3 genes mL^−1^ in the OMZ and ≤ 30 genes mL^−1^ in bathypelagic waters (Figure 3C).

The two variants of archaeal denitrification genes co-varied strongly with the two archaeal nitrification genes. Archaeal *nir*K-a gene abundance strongly correlated with archaeal *amo*A-HAC gene abundance (Figure S2A) and the abundance of archaeal *nir*K-b correlated with the *amo*A-LAC gene abundance (Figure S2B). Total archaeal *nir*K *vs*. total *amo*A (calculated as the sum of *nir*K-a and *nir*K-b, and *amo*A-HAC and *amo*A-LAC gene abundance, respectively) were significantly correlated (y = 2.38 × ^0.99^, *r* = 0.67, *p* < 0.001). Archaeal *nir*K-b gene distribution was related to archaeal 16S rRNA gene abundance (*r* = 0.60, *p* < 0.001). No significant (*r* ≥ 0.5, *p* ≤ 0.01) correlation between denitrification gene abundance and environmental parameters was found.

### Prokaryotic Community Composition

**Figure 4:**
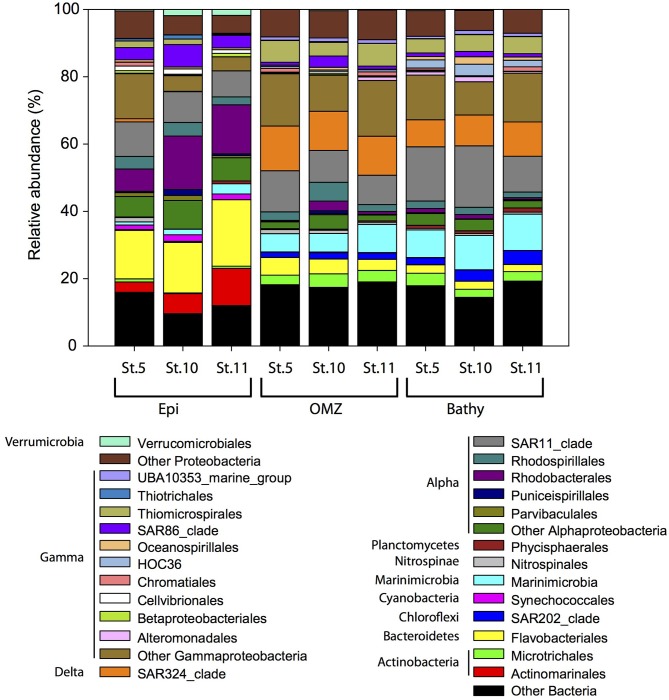
Relative abundance of major bacterial groups at the order level in specific stations and depth layers based on bacterial 16S rRNA gene sequencing. Orders that contribute ≥1% to the community are shown. Members of orders contributing ≤1% to the community are grouped under “Other Bacteria,” “Other Proteobacteria,” “Other Alphaproteobacteria,” and “Other Gammaproteobacteria.” Epi, epipelagic zone; OMZ, oxygen minimum zone; Bathy, bathypelagic zone; St., station.

Within the Bacteria domain, Alphaproteobacteria (Rhodobacterales, Rhodospirillales, and SAR11 clade) dominated throughout the water column (Figure 4), ranging between 40.9% in the epipelagic (station 10) and 13.6% in the bathypelagic (station 11). Bacteroidetes (Flavobacteriales) decreased with depth, from 20.7% in epipelagic waters (station 11) to 2.9% in bathypelagic waters (station 11). The abundance of Gammaproteobacteria increased with depth from 11.2% in the epipelagic to 28.7% in the OMZ and 26.4% in the bathypelagic (at station 11). Some Gammaproteobacteria orders (Alteromonadales, HOC36, Oceanospirillales, Thiomicrospirales) increased with depth, whereas SAR86 clade, Thiotrichales, Cellvibrionales, and Betaproteobacteriales decreased with depth. Within Actinobacteria, the Actinomarinales decreased with depth (Figure 4) from 11.1% in the epipelagic (station 11) to undetectable in the bathypelagic (station 5 and 10), whereas Microtrichales were more abundant at OMZ (4.0% at station 10) and bathypelagic (3.7% at station 5) than in epipelagic waters. Members of Verrucomicrobiales and Synechococcales (Cyanobacteria) were more abundant in epipelagic waters, up to 1.8 and 1.9%, respectively, at station 5. SAR324 clade (Deltaproteobacteria) (8.0–13.3%), members of the Phycisphaerales from Planctomycetes (up to 1.2%) and SAR202 (Chloroflexi) (up to ~4.1%) and Marinimicrobia (5.4–10.8%) contributed more to the bacterial communities in the OMZ and bathypelagic waters than to epipelagic waters, where SAR324, Phycisphaerales, and SAR202 contributed <0.9% and Marinimicrobia contributed 0.9–3.0%. The abundance of Betaproteobacteria was very low (≤1%) throughout the water column, and betaproteobacterial ammonia oxidizers ranged between undetectable and 0.06% in the 16S rRNA libraries. Nitrospinae bacteria, including NOB, accounted for up to 1.3% of the bacterial community composition in the epipelagic waters at station 5 (Figure 4).

*Candidatus* Brocadia (family Brocadiae) were the most abundant members of the anammox Bacteria throughout the water column (Table 1) ranging between 100 and 70% of the total anammox bacterial sequences, while *Candidatus* Scalindua ranged between 0 and 30% throughout the water column. The Planctomycetales order, to which Brocadia and Scalindua belong, represented between 0 and 0.3% of the 16S rRNA bacterial communities.

**Table 1:** Contribution of different archaeal families and groups and of different bacterial anammox genera to the archaeal and anammox communities, respectively.

		**16S rRNA archaea**										**16S rRNA anammox bacteria**	
	**Class**	**Thaumarchaeota**			**Nanoarchaeota**		**Thermoplasmata**			**Euryarchaeota**	**Archaea**	**Brocadiae**	**Brocadiae**
	**Fam**	**Nitrosopumilaceae**	**Unclass**.	**MBGA**	**Woesearchaeia**	**Unclass**.	**Marine group II**	**Marine group III**	**Halobacteria Marine group IV Halomicrobiaceae**	**Unclass**.	**Unclass**.	***Cand*. Brocadia**	***Cand*. Scalindua**
Epi	St. 5	57.90	0.00	0.00	0.00	0.00	40.84	0.86	0.01	0.06	0.32	100.00	0.00
	St. 10	–	–	–	–	–	–	–	–	–	–	–	–
	St. 11	43.34	0.05	0.00	0.15	0.00	55.43	0.60	0.00	0.04	0.38	84.39	15.61
OMZ	St. 5	66.94	0.02	0.21	0.38	0.00	27.59	4.10	0.04	0.46	0.25	69.96	30.04
	St. 10	54.31	0.08	0.35	0.66	0.01	38.85	5.00	0.03	0.14	0.57	–	–
	St. 11	62.75	0.09	0.33	0.33	0.02	31.74	4.32	0.11	0.00	0.30	87.59	12.41
Bathy	St. 5	60.99	0.22	0.43	0.69	0.00	30.10	6.66	0.04	0.20	0.67	–	–
	St. 10	64.21	0.00	0.54	1.52	0.00	26.59	6.50	0.21	0.15	0.22	–	–
	St. 11	63.25	0.08	0.35	1.00	0.00	29.03	5.93	0.05	0.02	0.29	76.16	23.84

*Bathy, bathypelagic zone; OMZ, oxygen minimum zone; Epi, epipelagic zone; St., station; MBGA, marine benthic group A, Unclass., Unclassified. Dashes indicate no data available*.

Members of the thaumarchaeal family Nitrosopumilaceae, belonging to Marine group I (MGI), were the dominant Archaea in the OMZ and bathypelagic waters (Table 1) ranging from 54.3 to 66.9% of total archaeal abundance. The abundance of members of Marine group II (MGII) Euryarchaeota decreased with depth from 40.8 to 55.4% in epipelagic waters to 26.6–30.1% in bathypelagic waters (Table 1). Members of the Marine group III (MGIII) Euryarchaeota increased from 0.6 to 0.9% in epipelagic waters to 5.9–6.7% in bathypelagic waters. Marine group IV (MGIV; Halobacteria) and Marine benthic group A (MBGA) were found in the OMZ and bathypelagic waters, ranging between 0.03–0.2% and 0.2–0.5%, respectively (Table 1). Members of Nanoarchaeota were more abundant at depth, reaching 1.5% at station 10.

Spearman's rank correlations of members of the prokaryotic community and the measured physico-chemical parameters are summarized in Table S4. Briefly, members of “other” Alphaproteobacteria and Verrucomicrobiales were positively correlated with nitrite (*r* ≥ 0.71, *p* ≤ 0.05). Flavobacteriales, Parvibaculales were positively correlated with ammonia concentrations (*r* ≥ 0.68, *p* ≤ 0.05; Figure S3). Members of SAR202 clade, Marinimicrobia, HOC36 correlated negatively only with ammonia concentrations (*r* ≤ −0.69, *p* ≤ 0.05). In contrast, members of SAR324, Alteromonadales, and UBA10353 marine group were negatively correlated with nitrite (*r* ≤ −0.71, *p* ≤ 0.01) and ammonia concentrations (*r* ≤ −0.69, *p* ≤ 0.05). Members of Deltaproteobacteria (SAR324), Gammaproteobacteria (Thiomicrospirales) and “other” Alphaproteobacteria were positively correlated with nitrate (*r* ≥ 0.71, *p* ≤ 0.05; Figure S3). In contrast, Actinobacteria, SAR86, and Verrumicrobiales were negatively correlated with nitrate concentrations (*r* ≤ −0.71, *p* ≤ 0.05). Members of Halobacteria, unclassified Nanoarchaeaeota, MBGA, and unclassified Thaumarchaeota were negatively correlated with nitrite (*r* ≤ −0.71, *p* ≤ 0.05). Halobacteria, Woesearchaeia, and MBGA correlated with ammonia concentrations negatively (*r* ≤ −0.73, *p* ≤ 0.05), whereas MGII correlated positively (*r* ≥ 0.67, *p* ≤ 0.05; Figure S3). No significant correlations were found between anammox members with nitrite, nitrate nor ammonia concentrations.

Archaeal richness and diversity indexes increased with depth (*p* ≤ 0.004, Kruskal–Wallis one-way ANOVA on ranks), while remaining fairly stable for Bacteria and anammox across the different depth layers (Table S5).

### Composition of Denitrifying and Ammonia Oxidizing Communities

**Figure 5:**
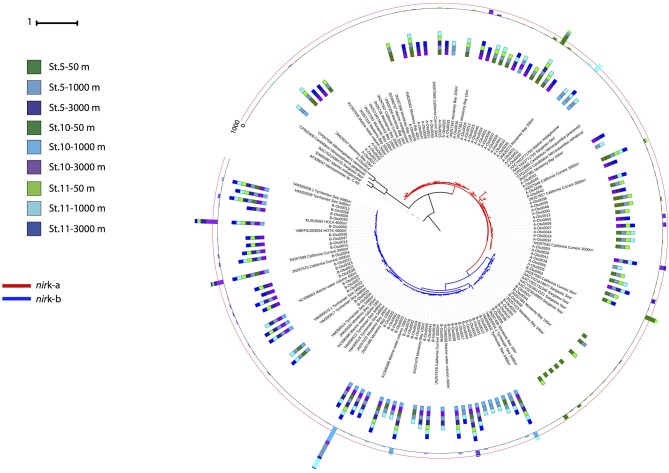
Phylogenetic tree of the 100 most abundant archaeal *nir*K OTUs type a and b sampled in the Gulf of Alaska. One representative of sequence group >97% identical is shown; the bar size shows the number of sequences represented by an OTU, and the colors indicate the depth layer. Green tones: epipelagic; light blue tones: oxygen minimum zone; dark blue tones: bathypelagic. Reference sequences as well as environmental sequences from archaeal *nir*K are included.

Phylogenetic analysis of the archaeal *nir*K genes revealed two main clusters for each of the two gene types (Figure 5). Three OTUs dominated the archaeal *nir*K-a community, A-Otu0001 and A-Otu0002 mainly included sequences from epipelagic and OMZ waters, while A-Otu0003 was present in bathypelagic waters and the OMZ. All representative archaeal *nir*K-a OTUs were closely related to the *Nitrosopumilus* genus *nir*K. Archaeal *nir*K-b revealed two main clusters dominated by five OTUs. One cluster consisted mainly of sequences from bathypelagic and OMZ waters (with very few sequences from epipelagic layers), while the other cluster consisted exclusively of epipelagic sequences. The OTUs from archaeal *nir*K-b were only closely related to environmental sequences from the oxygenated epi-, meso- and bathypelagic water of the open ocean and coastal environments but not to isolates (Figure 5).

Sequences of bacterial *nir*K genes were only successfully amplified in four samples (Figure S4). Phylogenetic analysis of the bacterial *nir*K showed a clear stratification with depth. Two OTUs, closely related to the alphaproteobacterial genus *Phaeobacter*, dominated the epipelagic waters (Otu0002 and Otu0003). The dominant bathypelagic *nir*K harboring Bacteria were closely related to the genera *Nitrosomonas* (Otu0001 and Otu0004) and *Pseudomonas* (Otu0006), or to environmental sequences (Otu0005, Otu0007, Otu0008, and Otu0009; Figure S4).

Archaeal and bacterial *nir*K richness and diversity generally increased with depth (Table S5). Archaeal *nir*K-b diversity parameters were significantly lower in epipelagic waters than in the OMZ (number of observed OTUs, *p* = 0.004; Shannon index, *p* = 0.032; Chao1 index, *p* = 0.007; Kruskal–Wallis ANOVA on ranks) and bathypelagic waters (Inverse Simpson index, *p* = 0.015).

**Figure 6:**
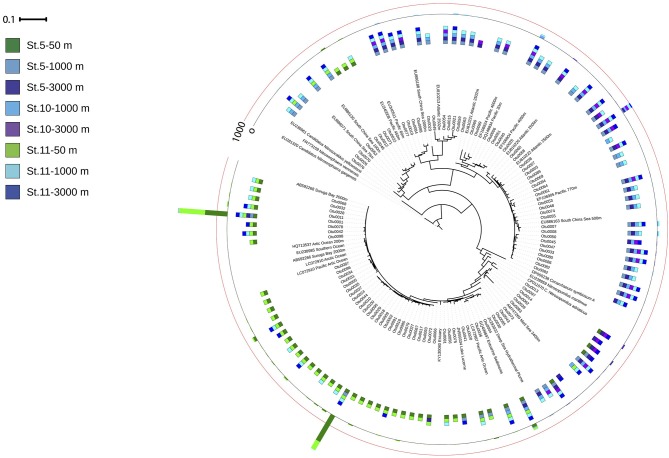
Phylogenetic tree of the 100 most abundant archaeal *amo*A OTUs recovered from the Gulf of Alaska. One representative of sequence group >97% identical is shown; the bar shows the number of sequences represented by an OTU, and the colors indicate the depth layer (Green tones: epipelagic; light blue tones: oxygen minimum zone; dark blue tones: bathypelagic). Reference sequences as well as environmental sequences from HAC- and LAC-amoA are included.

The *amo*A harboring community was dominated by three OTUs (Figure 6). The two most abundant OTUs (Otu0001 and Otu0002) were affiliated to the *amo*A-HAC ecotype, closely related to the *Nitrosopumilus* genus, and dominated in epipelagic waters. Two OTUs (Otu0003 and Otu0006) related to *amo*A-LAC ecotype dominated in deep waters, and were only related environmental sequences. The diversity of the ammonia oxidizing community (Inverse Simpson index) increased with depth, while richness (Chao1 index, number of observed OTUs) decreased with depth (Table S5). The number of OTUs found in epipelagic waters was significantly larger than in bathypelagic waters (*p* = 0.011, Kruskal–Wallis ANOVA on rank).

## Discussion

The eastern subtropical North Pacific is characterized by a pronounced OMZ at 700–1,300 m depth, with oxygen concentration below 20 μM in its core. Currently, this OMZ seasonally extends northwards into the Gulf of Alaska (Paulmier and Ruiz-Pino, 2009), being more intense in spring and fall. It is forecasted that OMZs are expanding and intensifying as a response to anthropogenic impacts (Stramma et al., 2008; Keeling et al., 2010). OMZs are hotspots of microbial activity and particularly of processes involved in the nitrogen cycle (Lam and Kuypers, 2011). Microbial players of the nitrogen cycle have been thoroughly studied in permanent open ocean OMZs, such as the Arabian Sea and the eastern Tropical South Pacific (Pitcher et al., 2011; De Brabandere et al., 2014) and shallow seasonal OMZs (Molina et al., 2010; Galan et al., 2012). Seasonal OMZs, characterized by alternation of environmental conditions stimulate or inhibit several processes involved in the nitrogen cycle (Galan et al., 2017). These seasonal OMZs are particularly suited to study the coupling and dynamics between different microbial players in the nitrogen cycling and to understand the microbial response to expanding OMZs (Wright et al., 2012; Hallam et al., 2017).

### Depth Distribution of Bacterial and Archaeal Phylotypes

The relative abundance of Thaumarchaeota in the Gulf of Alaska increased in meso- and bathypelagic waters, while the relative bacterial abundance decreased with depth in agreement with previous studies in the Pacific, Atlantic, and the Mediterranean Sea (Agogue et al., 2008; De Corte et al., 2009; Church et al., 2010; Santoro et al., 2010).

Overall, the bacterial community composition in the Gulf of Alaska exhibited a similar depth stratification in relation to various environmental parameters as previously reported for the Atlantic and Pacific (Delong et al., 2006; Treusch et al., 2009; Agogue et al., 2011). Briefly, the epipelagic waters are inhabited mainly by Rhodobacterales and Flavobacteriales previously reported to respond to phytoplankton blooms (Teeling et al., 2012, 2016; Taylor et al., 2014). The OMZ and bathypelagic waters are characterized by an increase in the contribution of chemoautotrophs such as SAR324, and Planctomycetes, to which anammox Bacteria belong, as well as groups involved in the sulfur cycle (e.g., Marinimicrobia, Thiotrichales, Thiomicrospirales), similarly to coastal oxygen-depleted environments (Aldunate et al., 2018). These groups correlated negatively with oxygen concentrations (Figure S3) and might benefit from gradients in oxygen and redox potential in the OMZ providing different electron acceptors and donors for microbial metabolism (Stevens and Ulloa, 2008).

The archaeal community is dominated by MGI Thaumarchaeota (Nitrosopumilaceae) and MGII Euryarchaeota followed by MGIII Euryarchaeota, Nanoarchaeota, Marine Benthic Group A, and members of the Halobacteria (MGIV). MGI Thaumarchaeota and MGII Euryarchaeota have been reported to be the dominant groups of the marine archaeal community from epipelagic (Karner et al., 2001; Martin-Cuadrado et al., 2015) to bathypelagic waters (López-García et al., 2001; Teira et al., 2006; Li et al., 2015). In contrast, MGIII and MGIV Euryarchaeota are present in low abundance in bathypelagic communities (Fuhrman and Davis, 1997; Galand et al., 2009).

### Potential for Nitrification and Denitrification of Archaeal Ecotypes

Thaumarchaeota have been estimated to represent ~20% of the prokaryotic cells in the ocean (Karner et al., 2001), however, they are present in only low abundance in open ocean surface waters (Tolar et al., 2016b). Besides light inhibition (Merbt et al., 2012), it has been suggested that Thaumarchaeota are inhibited by hydrogen peroxide (H_2_O_2_), a by-product of photochemical and biological processes in surface waters (Tolar et al., 2016a). Thaumarchaeal cells lack detoxifying enzymes for this highly reactive compound (Tolar et al., 2016b; Bayer et al., 2019a). Additionally, other environmental factors such as ammonia concentration (Sintes et al., 2013) and temperature (Groussin and Gouy, 2011) support the niche differentiation of marine Thaumarchaeota into two ecotypes (Francis et al., 2005; Beman et al., 2008; Sintes et al., 2013, 2016; Luo et al., 2014). The two ecotypes of ammonia oxidizing Archaea, HAC- and LAC-AOA, showed a depth distribution in the Gulf of Alaska similar to that previously reported for the Atlantic (Sintes et al., 2016) and Pacific (Santoro et al., 2017). HAC-AOA dominates in the epipelagic, characterized by micromolar concentrations of ammonium (Table S1), and LAC-AOA in meso- and bathypelagic waters, where ammonium concentrations are in the nanomolar range to below the detection limit (Table S1). These data are in agreement with the findings of Sintes et al. (2013, 2016), Smith et al. (2016), and Santoro et al. (2017) who suggested that these ecotypes are adapted to different ammonium concentrations and supply rates. The location of the OMZ, at around 1,000 m depth, coincides with the increase in the LAC-AOA, as indicated in previous studies in other shallow OMZs (Bertagnolli and Ulloa, 2017). The deeper location of the OMZ in the Gulf of Alaska results in a larger dominance of the LAC-AOA, which comprised 90.9 ± 5.1% of the AOA community as compared with 42% in shallower OMZs (Bertagnolli and Ulloa, 2017), suggesting that oxygen concentration is not the main driver of the distribution of these two ecotypes. However, the environmental factors behind this depth distribution pattern remain unknown.

Notably, the two variants of thaumarchaeal *nir*K genes (*nir*K-a and *nir*K-b) also showed a distinct depth distribution pattern (Figure 3), in agreement with previous findings from the Pacific off Monterey Bay and from the California Current (Lund et al., 2012). Archaeal *nir*K-a containing cells dominate in epi- and upper mesopelagic waters while archaeal *nir*K-b containing cells dominate in meso- and bathypelagic waters, similar to previous findings (Lund et al., 2012). This distribution pattern tentatively suggests that archaeal *nir*K-a is mainly found in the HAC-*amo*A containing cells, and that archaeal cells containing *nir*K-b are also harboring LAC-*amo*A. This notion is further supported by the close affiliation of *nir*K-a and *amo*A-HAC to *nir*K and *amo*A gene from *Nitrosopumilus maritimus, Cand*. Nitrosopumilus piranensis, and *Cand*. Nitrosopumilus adriaticus, while archaeal *nir*K-b and *amo*A-LAC sequences are solely associated with environmental sequences (Figures 5, 6). Moreover, the covariation of *nir*K-a and HAC-*amo*A abundance, on the one hand, and *nir*K-b and LAC-*amo*A abundance, on the other hand, explain the variation in archaeal and bacterial communities over the depth profile in the Gulf of Alaska OMZ (Figure S3). Collectively, these findings reveal that the AOA ecotypes not only vary in their ammonia monooxygenase, but also in their nitrite reductase (Figure S3). Furthermore, this indicates a resource-based niche-partitioning not only based on the affinity to ammonia (Sintes et al., 2013; Smith et al., 2016), but also by their potential to use alternative sources of energy (Qin et al., 2014; Smith et al., 2016). The potentially wider substrate range of LAC-AOA (Water Cluster B, WCB) is further supported by the mismatch between their relatively high abundance in deep waters and the low nitrification rates at these depths, which otherwise would require an extremely long turnover time based on ammonia chemoautotrophy (Newell et al., 2011; Smith et al., 2016). All cultured isolates of AOA (Könneke et al., 2005; Santoro et al., 2015; Bayer et al., 2019b) belong to the HAC-AOA (or water cluster A, WCA), proposed to be mostly obligate chemoautotrophs. However, less is known about LAC-AOA, which have eluded cultivation thus far. Some AOA can use alternative energy sources such as urea (Bayer et al., 2019b) for inorganic carbon fixation, or could exhibit mixotrophic or heterotrophic lifestyles. Potential mixotrophy or heterotrophy have been suggested for Thaumarchaeota cells based on the observation of organic acids incorporation by Archaea (Varela et al., 2008; Clifford et al., 2019) and expression of transporter proteins for organic acids (Bergauer et al., 2018). Recent findings also indicate differences in ecological interactions of the two ecotypes. Preferential association of bacterial phylotypes with one of the AOA ecotypes has been described, such as the specific association between Nitrospina and members of HAC (WCA-like cluster; Reji et al., 2019). This linkage supports a major role of the HAC-ecotype in nitrification (Smith et al., 2014) suggesting that reciprocal feeding between NOB and AOA (Pachiadaki et al., 2017) would be predominantly between NOB and HAC-AOA, with implications for the nitrogen cycle in zones where LAC-AOA are dominant, such as in the deep ocean OMZ. However, further research is needed to elucidate the contribution of heterotrophic or mixotrophic Thaumarchaeota in the ocean.

The role of the nitrite reductase in aerobic ammonia oxidizers is still unclear. Copper containing nitrite reductase, *nir*K, has been found in both, bacterial and archaeal ammonia-oxidizers (Casciotti and Ward, 2001; Treusch et al., 2005; Bartossek et al., 2010; Lund et al., 2012). It has been suggested that the *nir*K enables bacterial ammonia oxidizers to tolerate environments with high ammonia and low O_2_ concentrations (Kozlowski et al., 2014). Recently, a three-step ammonia oxidation pathway was proposed for Bacteria (Caranto and Lancaster, 2017) with two obligate intermediates, hydroxylamine (NH_2_OH) and nitric oxide (NO). In this proposed pathway, *nir*K would catalyze the oxidation of NO produced by hydroxylamine oxidoreductase (HAO) in ammonia-oxidizing Bacteria (AOB) to form ${\text{NO}}_{2}^{-}$ (Caranto and Lancaster, 2017). Despite the absence of a HAO homolog in Thaumarchaeota (Kerou et al., 2016), the production of these two intermediates in AOA (Vajrala et al., 2013; Kozlowski et al., 2016) suggests that ammonia oxidation in Archaea might also occur via a three-step process (Carini et al., 2018). The reported accumulation of N_2_O in this zone (Grundle et al., 2012) implies active nitrification/denitrification processes regardless of the low abundance of Bacteria involved in the nitrogen cycle, thus suggesting an important role of AOA.

### Bacterial Players on the Nitrogen Cycle in the Gulf of Alaska

Bacterial ammonia oxidizers, including *Nitrosomonas* and Nitrosococcales, were only present at very low abundances in the 16S rRNA libraries (0–0.06%), suggesting a minor role of bacterial nitrifiers throughout the water column of the Gulf of Alaska. Nitrite oxidizing Bacteria only accounted for ~1% of the bacterial community, in contrast to other OMZs (Fussel et al., 2012).

Denitrifying Bacteria include AOB, which under oxygen-limited conditions can substitute O_2_ with ${\text{NO}}_{2}^{-}$ as an alternative electron acceptor in the “nitrifier denitrification” pathway (Stein, 2011). However, other members of the Bacteria are capable of denitrification. The alphaproteobacterium *Phaeobacter gallaeciensis* is the closest cultured relative to the two most abundant epipelagic OTUs of denitrifying Bacteria in this study. *Phaeobacter gallaeciencis* is adapted to colonize surfaces (Thole et al., 2012; Freese et al., 2017). The distribution of bacterial *nir*K in the Gulf of Alaska does not seem to be related to dissolved oxygen concentrations. However, marine snow and the zooplankton gut provide diffusion limited microenvironments where aerobic respiration results in oxygen depletion (Alldredge and Cohen, 1987) and thus, expand the niche of anoxic metabolism such as denitrification to otherwise well oxygenated ocean waters (Dang and Lovell, 2016; Bianchi et al., 2018). Ganesh et al. (2015) reported an enrichment of transcripts of genes related to denitrification in particle-associated communities relative to free-living communities in the OMZ. In addition, AOB, such as *Nitrosomonas eutropha* (Betaproteobacteria), a close relative to the dominant denitrifier OTUs in the bathypelagic realm in this study (Figure S4), have been related to a particle-associated life style (Phillips et al., 1999). Marine snow could provide not only an oxygen-depleted microenvironment but also a regular supply of ammonia associated to the decomposition of organic material (Thole et al., 2012). The particle-associated lifestyle of the denitrifying Bacteria could help explaining their low abundance in our samples (<33 *nir*K genes mL^−1^) and their increase in the vicinity to the seafloor where resuspension might introduce particles into the water column. However, the bacterial denitrifiers abundance and diversity in the Gulf of Alaska are most probably underestimated, as we could not successfully amplify the *nir*S-containing organisms, which have been reported as abundant and diverse nitrifiers (Dang et al., 2009; Jayakumar et al., 2013).

### Anaerobic Ammonia Oxidizers

Anammox Bacteria were present in low abundance in the Gulf of Alaska, consisting of *Candidatus* Brocadia followed by *Candidatus* Scalindua. This is in contrast to previous observations in marine and freshwater environments where *Cand*. Scalindua was found to be relatively abundant (Penton et al., 2006; Schmid et al., 2007; Van De Vossenberg et al., 2008; Woebken et al., 2008; Villanueva et al., 2014). *Cand*. Brocadia has previously been reported as the dominant anammox bacterium in waste water treatment plants (Taylor, 2012; Taylor et al., 2014) corresponding to the general preference of anammox Bacteria to environments with high ${\text{NH}}_{4}^{+}$ and ${\text{NO}}_{2}^{-}$ concentrations (Oshiki et al., 2016). Anammox bacteria can be active at low levels of O_2_ ranging between 1 and ~10 μmol O_2_ L^−1^ (Jensen et al., 2011) and up to ~20 μmol O_2_ L^−1^ (Kalvelage et al., 2011; Dalsgaard et al., 2014). Bristow et al. (2016) demonstrated anammox activity at O_2_ levels as low as 5–30 nM using a highly sensitive oxygen sensor. However, the minimum O_2_ concentration we measured in our study was 11.8 μmol L^−1^ thus, in the upper range of the oxygen concentration for anammox activity. Although 16S rRNA genes affiliated to anammox Bacteria were detected in the GoA waters in agreement with previous findings in zones with low but measurable oxygen concentrations (Dalsgaard et al., 2012), anammox Planctomycetes are highly oxygen-sensitive.

Taken together, our results support the broad distribution of archaeal ammonia oxidizers in the ocean's OMZs and the diversity of microbial niches present in OMZs (Bertagnolli and Stewart, 2018). Bacterial denitrifiers harboring the *nir*K gene are present throughout the water column potentially exhibiting a particle-associated life style, which can provide anoxic or microaerobic microenvironments facilitating oxygen-sensitive metabolisms (Dang and Lovell, 2016). This niche-specific association explains the generally low abundance of bacterial denitrifiers due to the patchiness of marine snow (Silver et al., 1978), and their relatively higher abundances in the epipelagic realm, where marine snow is formed by phytoplankton activity and their exudates, and in bathypelagic waters, where aggregation can lead to the formation of large particles (Bochdansky et al., 2016). Anammox Bacteria are confined to an even narrower niche than denitrifying Bacteria. Their low abundance throughout the water column suggests that they are members of the rare biosphere (Sogin et al., 2006) and potentially, members of a seed bank (Lennon and Jones, 2011), ready to increase in abundance once favorable environmental conditions are established, i.e., the intensification and areal expansion of the OMZ during the fall in this area. Consequently, archaeal ammonia oxidizers play a major role in the nitrogen cycle throughout the water column in the Gulf of Alaska. However, AOA display a distinct ecotype distribution with *amo*A-HAC/*nir*K-a and *amo*A-LAC/*nir*K-b dominating in epi- and meso- to bathypelagic waters, respectively. Archaeal ammonia oxidizing ecotypes differ not only in their *amo*A gene but also in their *nir*K gene, suggesting that this niche separation implicates a profound change in the substrate preferences of these ecotypes.

## Author Contributions

ES and GH designed the work. SM, DD, and EC performed the research. BB provided *Nitrosopumilus* cultures. SM, DD, and ES performed the data analysis. SM, DD, GH, and ES wrote the study. All coauthors approved the final version of the manuscript.

### Conflict of Interest Statement

The authors declare that the research was conducted in the absence of any commercial or financial relationships that could be construed as a potential conflict of interest.

## Supplementary Material

Table S1 Environmental parameters measured in the different stations sampled. Pot. Temp., potential temperature; AOU, apparent oxygen utilization. Dashes indicate no data available.

Table S2 Primer sets and amplification parameters used for the phylogenetic and functional marker genes amplification by qPCR. HAC, “high-ammonia concentration” archaeal *amo*A gene; LAC, “low-ammonia concentration” archaeal *amo*A gene; Thau, Thaumarchaeota; *nir*K-a and *nir*K-b, archaeal nitrate reductase K type a and type b, respectively.

Table S3 Primer sets and cycling conditions used to amplify bacterial, archaeal, and anammox phylogenetic and functional genes for next-generation sequencing.

Table S4 Spearman's rank correlation coefficients between the different bacterial and archaeal groups and the environmental parameters measured. Only significant results of the bacterial and archaeal community are shown (−0.5 > rs > 0.5). No significant correlations were found for anammox bacteria. Numbers in bold are *p* ≤ 0.01. Dashes indicate no significant correlation for the specific parameter. AOU, Apparent oxygen utilization; Pot. Temp., potential temperature.

Table S5 Diversity parameters for phylogenetic (based on 16S rRNA) and functional prokaryotic groups. nitrif., nitrification; *amo*A, archaeal *amo*A genes; *nir*K-a and *nir*K-b, archaeal nitrite reductase K variants a and b; bac *nir*K, bacterial nitrite reductase K; epi, epipelagic zone; OMZ, oxygen minimum zone; bathy, bathypelagic zone; nseqs, number of sequences sampled for OTU definition; inv. Simpson, inverse of the Simpson index; sobs, number of OTUs observed. Numbers in bold are significantly different (Kruskal–Wallis ANOVA on rank, *p* < 0.004).

Dataset S1 Average gene abundances and standard deviation determined by q-PCR.

Figure S1 Salinity-temperature diagram of the different stations sampled in the Gulf of Alaska **(A)**. Depth profiles of dissolved oxygen **(B)**, dissolved organic carbon **(C)**, and ammonia **(D)** concentration in different stations.

Figure S2 Correlation between archaeal denitrification (*nir*K-a, *nir*K-b) and nitrification (*amo*A HAC, *amo*A LAC) genes. **(A)** “High-ammonia concentration” archaeal *amo*A (*amo*A HAC) vs. archaeal nitrate reductase K variant a (*nir*K-a); **(B)** “low-ammonia concentration” archaeal *amo*A (*amo*A LAC) vs. archaeal nitrate reductase K variant b (*nir*K-b).

Figure S3 Canonical correspondence analysis of the bacterial and archaeal phylotypes using the environmental parameters and the phylogenetic and functional gene abundance.

Figure S4 Phylogenetic tree of the 100 most abundant bacterial *nir*K OTUs sampled in the Gulf of Alaska. One representative of each sequence group >97% identical is shown; the bar shows the number of sequences represented by the OTU, and the color indicates the depth layer. Green tones: epipelagic; dark blue tones: bathypelagic. Reference and environmental sequences are included.
