# Supplementary material for: Niche Differentiation of Aerobic and Anaerobic Ammonia Oxidizers in a High Latitude Deep Oxygen Minimum Zone
Source: Front Microbiol. 2019 Sep 13;10:2141. doi: 10.3389/fmicb.2019.02141 (PMC6753893; doi:10.3389/fmicb.2019.02141)
Supplement: Table S1 — Environmental parameters measured in the different stations sampled. Pot. Temp., potential temperature; AOU, apparent oxygen utilization. Dashes indicate no data available. [file Table_1.DOCX]

**Table S1.** Environmental parameters measured in the different stations sampled. Abbreviations: Pot. Temp., potential temperature; AOU, apparent oxygen utilization. Dashes indicate no data available.

| Station | Latitude  (N) | Longitude (W) | Depth  (m) | Pot. temp.  (°C) | Salinity | Oxygen  (µmol kg^-1^) | AOU  (µmol kg^-1^) | Nitrite  (µmol kg^-1^) | Nitrate  (µmol kg^-1^) | Ammonia  (µmol kg^-1^) |
| --- | --- | --- | --- | --- | --- | --- | --- | --- | --- | --- |
| 1 | 52.56 | 141.32 | 3 | 14.62 | 32.48 | 281.40 | -27.99 | - | - | - |
| 1 | 52.56 | 141.32 | 49 | 5.98 | 32.65 | 278.90 | 27.23 | 0.46 | 15.65 | 0.11 |
| 1 | 52.56 | 141.32 | 1005 | 3.02 | 34.37 | 14.00 | 311.91 | 0.02 | 45.52 | 0.01 |
| 1 | 52.56 | 141.32 | 2024 | 1.93 | 34.59 | 56.30 | 278.79 | 0.03 | 42.13 | 0.01 |
| 1 | 52.56 | 141.32 | 3048 | 1.58 | 34.66 | 106.50 | 232.67 | 0.02 | 38.05 | 0.07 |
| 1 | 52.56 | 141.32 | 3853 | 1.50 | 34.68 | 127.10 | 213.09 | 0.02 | 37.09 | 0.00 |
| 2 | 54.35 | 141.24 | 4 | 14.18 | 32.52 | 282.10 | -26.43 | 0.12 | 8.97 | 0.04 |
| 2 | 54.35 | 141.24 | 49 | 5.87 | 32.65 | 298.40 | 8.54 | 0.39 | 18.43 | 0.52 |
| 2 | 54.35 | 141.24 | 1012 | 3.00 | 34.37 | 16.00 | 310.09 | 0.01 | 44.01 | 0.29 |
| 2 | 54.35 | 141.24 | 2026 | 1.96 | 34.58 | 54.90 | 280.02 | 0.01 | 42.08 | 0.00 |
| 2 | 54.35 | 141.24 | 3050 | 1.59 | 34.66 | 110.10 | 228.41 | 0.02 | 38.48 | 0.00 |
| 2 | 54.35 | 141.24 | 3718 | 1.48 | 34.68 | 139.20 | 200.88 | 0.01 | 40.51 | 0.48 |
| 3 | 56.28 | 141.14 | 3 | 15.84 | 32.27 | 270.40 | -22.85 | 0.01 | 0.23 | 2.53 |
| 3 | 56.28 | 141.14 | 47 | 5.95 | 32.67 | 298.70 | 7.64 | 0.56 | 15.38 | 1.4 |
| 3 | 56.28 | 141.14 | 1010 | 3.07 | 34.38 | 22.00 | 303.56 | 0.01 | 43.8 | 0.36 |
| 3 | 56.28 | 141.14 | 2025 | 1.91 | 34.59 | 58.00 | 277.29 | 0.01 | 41.58 | 0.49 |
| 3 | 56.28 | 141.14 | 3049 | 1.57 | 34.66 | 111.40 | 227.45 | 0.01 | 39.08 | 0.11 |
| 3 | 56.28 | 141.14 | 3631 | 1.46 | 34.68 | 140.70 | 199.60 | 0.01 | 37.96 | 1.14 |
| 4 | 58.59 | 140.59 | 4 | 15.39 | 31.48 | 291.60 | -40.60 | 0.02 | 0.08 | 1.2 |
| 4 | 58.59 | 140.59 | 49 | 6.48 | 32.30 | 244.60 | 58.67 | 0.02 | 20.54 | 1.08 |
| 5 | 57.09 | 148.42 | 5 | 13.20 | 32.50 | 290.20 | -29.83 | 0.11 | 5.1 | 3.03 |
| 5 | 57.09 | 148.42 | 51 | 5.46 | 32.67 | 282.70 | 27.21 | 0.03 | 19.4 | 1.02 |
| 5 | 57.09 | 148.42 | 1011 | 2.92 | 34.38 | 16.40 | 310.33 | 0.01 | 43.7 | 0.28 |
| 5 | 57.09 | 148.42 | 2028 | 1.89 | 34.59 | 61.30 | 274.22 | 0.01 | 40.93 | 0.79 |
| 5 | 57.09 | 148.42 | 3049 | 1.59 | 34.66 | 113.00 | 225.71 | 0.01 | 39.01 | 0.37 |
| 5 | 57.09 | 148.42 | 3941 | 1.44 | 34.69 | 152.40 | 188.36 | - | - | - |
| 6 | 54.60 | 152.39 | 4 | 12.64 | 32.34 | 258.40 | 6.01 | 0.05 | 2.83 | 0.68 |
| 6 | 54.60 | 152.39 | 48 | 4.85 | 32.85 | 277.30 | 34.40 | 0.01 | 22.85 | 0.32 |
| 6 | 54.60 | 152.39 | 1012 | 2.77 | 34.41 | 11.80 | 316.09 | 0.01 | 38.88 | 0.06 |
| 6 | 54.60 | 152.39 | 2028 | 1.81 | 34.61 | 67.40 | 268.71 | 0.00 | 40.35 | 0.92 |
| 6 | 54.60 | 152.39 | 3049 | 1.50 | 34.67 | 124.80 | 214.70 | 0.00 | 33.35 | 0.34 |
| 6 | 54.60 | 152.39 | 4275 | 1.49 | 34.69 | 144.00 | 196.70 | 0.00 | 36.56 | 3.2 |
| 7 | 52.59 | 152.00 | 5 | 12.17 | 32.48 | 268.50 | -1.85 | 0.13 | 8.25 | 1.26 |
| 7 | 52.59 | 152.00 | 51 | 5.12 | 32.86 | 283.30 | 28.77 | 0.05 | 24.89 | 0.93 |
| 7 | 52.59 | 152.00 | 1011 | 2.70 | 34.42 | 16.50 | 311.93 | 0.00 | 43.62 | 1.27 |
| 7 | 52.59 | 152.00 | 2024 | 1.87 | 34.60 | 59.70 | 275.90 | 0.00 | 41.32 | 1.06 |
| 7 | 52.59 | 152.00 | 3047 | 1.61 | 34.65 | 102.60 | 235.72 | 0.01 | 38.12 | 0.58 |
| 7 | 52.59 | 152.00 | 3558 | 1.49 | 34.68 | 133.20 | 206.81 | 0.00 | 37.37 | 0.35 |
| 8 | 51.00 | 152.00 | 4 | 12.74 | 32.46 | 271.60 | -8.13 | 0.13 | 10.24 | 0.41 |
| 8 | 51.00 | 152.00 | 49 | 6.04 | 32.62 | 287.20 | 18.58 | 0.89 | 17.62 | 0.65 |
| 8 | 51.00 | 152.00 | 1010 | 2.81 | 34.40 | 16.70 | 310.86 | 0.01 | 45.47 | 0.68 |
| 8 | 51.00 | 152.00 | 2031 | 1.90 | 34.59 | 55.20 | 280.21 | 0.01 | 42.83 | 0.08 |
| 8 | 51.00 | 152.00 | 3048 | 1.57 | 34.66 | 106.20 | 232.59 | 0.01 | 39.38 | 0.00 |
| 8 | 51.00 | 152.00 | 5079 | 1.59 | 34.69 | 142.90 | 196.68 | 0.04 | 37.11 | 0.52 |
| 9 | 49.59 | 144.59 | 4 | 14.62 | 32.36 | 225.10 | 28.47 | - | - | - |
| 9 | 49.59 | 144.59 | 50 | 6.43 | 32.57 | 260.50 | 42.54 | 0.31 | 13.82 | 0.44 |
| 9 | 49.59 | 144.59 | 1262 | 2.53 | 34.46 | 23.60 | 302.48 | - | - | - |
| 9 | 49.59 | 144.59 | 2025 | 1.93 | 34.58 | 53.70 | 281.49 | 0.00 | 41.53 | 1.62 |
| 9 | 49.59 | 144.59 | 3048 | 1.56 | 34.66 | 106.40 | 232.53 | 0.00 | 37.78 | 0.25 |
| 9 | 49.59 | 144.59 | 4316 | 1.52 | 34.68 | 136.20 | 204.25 | 0.00 | 36.1 | 1.14 |
| 10 | 49.48 | 141.47 | 4 | 15.16 | 32.37 | 279.00 | -28.18 | 0.11 | 6.85 | 0.12 |
| 10 | 49.48 | 141.47 | 40 | 8.10 | 32.55 | 278.00 | 13.61 | - | - | - |
| 10 | 49.48 | 141.47 | 1010 | 2.96 | 34.37 | 16.10 | 310.32 | 0.00 | 44.99 | 0.01 |
| 10 | 49.48 | 141.47 | 2025 | 1.92 | 34.59 | 58.50 | 276.77 | 0.03 | 41.16 | 0.01 |
| 10 | 49.48 | 141.47 | 3048 | 1.57 | 34.66 | 107.90 | 231.01 | 0.01 | 37.73 | 0.01 |
| 10 | 49.48 | 141.47 | 4096 | 1.52 | 34.68 | 126.40 | 213.85 | 0.01 | 36.59 | 0.01 |
| 11 | 49.25 | 136.39 | 5 | 16.25 | 32.37 | 225.70 | 19.74 | 0.09 | 2.16 | 1.1 |
| 11 | 49.25 | 136.39 | 50 | 8.91 | 32.42 | 245.90 | 40.60 | 0.32 | 7.16 | 1.55 |
| 11 | 49.25 | 136.39 | 1012 | 3.08 | 34.38 | 15.60 | 309.83 | 0.00 | 43.65 | 0.05 |
| 11 | 49.25 | 136.39 | 2026 | 1.93 | 34.59 | 53.00 | 282.11 | 0.00 | 41.48 | 0.3 |
| 11 | 49.25 | 136.39 | 3047 | 1.60 | 34.66 | 95.30 | 243.37 | 0.00 | 38.73 | 0.09 |
| 11 | 49.25 | 136.39 | 3887 | 1.55 | 34.67 | 111.00 | 228.80 | 0.00 | 37.59 | 0.4 |
| 12 | 49.16 | 134.40 | 4 | 16.19 | 32.41 | 236.20 | 9.46 | 0.08 | 3.89 | 0.37 |
| 12 | 49.16 | 134.40 | 50 | 8.07 | 32.54 | 297.70 | -5.90 | 0.16 | 10.59 | 0.63 |
| 12 | 49.16 | 134.40 | 1011 | 3.06 | 34.36 | 22.50 | 303.12 | 0.00 | 46.47 | 0.01 |
| 12 | 49.16 | 134.40 | 2023 | 1.96 | 34.59 | 66.20 | 268.82 | 0.00 | 42.45 | 0.03 |
| 12 | 49.16 | 134.40 | 3048 | 1.60 | 34.66 | 115.90 | 222.82 | 0.00 | 38.59 | 0.02 |
| 12 | 49.16 | 134.40 | 3681 | 1.58 | 34.67 | 126.80 | 212.81 | 0.00 | 37.5 | 0.01 |
