# Supplementary material for: Niche Differentiation of Aerobic and Anaerobic Ammonia Oxidizers in a High Latitude Deep Oxygen Minimum Zone
Source: Front Microbiol. 2019 Sep 13;10:2141. doi: 10.3389/fmicb.2019.02141 (PMC6753893; doi:10.3389/fmicb.2019.02141)
Supplement: Table S2 — Primer sets and amplification parameters used for the phylogenetic and functional marker genes amplification by qPCR. HAC, “high-ammonia concentration” archaeal amoA gene; LAC, “low-ammonia concentration” archaeal amoA gene; Thau, Thaumarchaeota; nirK-a and nirK-b, archaeal nitrate reductase K type a and type b, respectively. [file Table_2.DOCX]

| **Process** | **Target taxon** | **Gene*** | **Primer set** | **Sequence** | **Annealing temperature (°C)** | **Fragment length**  **(bp)** | **Reference** |
| --- | --- | --- | --- | --- | --- | --- | --- |
|  | Bacteria | *rec*A ^a, g^ | recA F | 5‘-GCI TTY ATY GAT GCI GAR CA-3‘ | 53 | 209 | (Holmes et al., 2004) |
|  |  |  | recA R | 5‘-CCC ATI TCI CCT TCD ATY TC-3‘ |  |  |  |
|  | Archaea (Thau) | 16S rRNA ^b, h^ | GI 751F | 5‘-GTC TAC CAG AAC AYG TTC-3‘ | 58 | 205 | (Mincer et al., 2007) |
|  |  |  | GI 956R | 5‘-HGG CGT TGA CTC CAA TTG-3‘ |  |  |  |
|  | Anammox | 16S rRNA ^c, i^ | Brod 541F | 5‘-GAG CAC GTA GGT GGG TTT GT-3‘ | 59 | 280 | (Li et al., 2010) |
|  |  |  | Amx 820R | 5‘-AAA ACC CCT CTA CTT AGT GCC C-3‘ |  |  |  |
| Nitrification | Archaea (HAC) | *amo*A ^d, i^ | archAmoA-for | 5'-CTG AYT GGG CYT GGA CAT C-3' | 59 | 256 | (Wuchter et al., 2006) |
|  |  |  | archAmoA-rev | 5'-TTC TTC TTT GTT GCC CAG TA-3' |  |  |  |
|  | Archaea (LAC) | *amo*A ^d, i^ | archAmoA-for | 5'-CTG AYT GGG CYT GGA CAT C-3' | 59 | 256 | (Wuchter et al., 2006) |
|  |  |  | archAmoA-rev New | 5’-TTC TTC TTC GTC GCC CAA TA-3’ |  |  | (Sintes et al., 2013) |
| Denitrification | Bacteria | *nir*K ^e, j^ | nirK-q-F | 5‘-TCA TGG TGC TGC CGC GYG A-3‘ | 68-60 | 472 | (Mosier and Francis, 2010) |
|  |  |  | nirK-1040 | 5‘-GCC TCG ATC AGR TTR TGG TT-3‘ |  |  |  |
|  | Archaea | *nir*K-a ^f, k^ | anirK-a 58F | 5‘-ACB YTA TTC GGA AGY ACA TAC ACA-3‘ | 50 | 521 | (Lund et al., 2012) |
|  |  |  | anirK-a 579R | 5‘-GYM ATT CCG TAC ATK CCG GA-3‘ |  |  |  |
|  | Archaea | *nir*K-b ^f, k^ | anirK-b 61F | 5‘-CTA TTC GGA RGT WCT TTY ACT GC-3‘ | 50 | 494 | (Lund et al., 2012) |
|  |  |  | anirK-b 555R | 5‘-ACG TGT TGG TCC ATT GCT GC-3‘ |  |  |  |

**Table S2.** Primer sets and amplification parameters used for the phylogenetic and functional marker genes amplification by qPCR. Abbreviations: HAC, ‘high-ammonia concentration’ archaeal *amo*A gene; LAC, ‘low-ammonia concentration’ archaeal *amo*A gene; Thau, Thaumarchaeota; *nir*K-a and *nir*K-b, archaeal nitrate reductase K type a and type b, respectively.

*Amplification parameters for q-PCR: **a**, 94 °C for 4 min, 35 x (94 °C for 1 min, 53 °C for 1 min, 72 °C for 7 min) followed by 72 °C for 7 min; **b**, 94 °C for 4 min, 35 x (94 °C for 1 min, 58 °C for 1 min, 72 °C for 1 min) followed by 72 °C for 7 min; **c**, 95 °C for 3 min, 35 x (95 °C for 45 sec, 59 °C for 1 min, 72 °C for 1 min) followed by 72 °C for 7 min; **d**, 95 °C for 4 min, 30 x (94 °C for 30 sec, 59 °C for 20 sec, 72 °C for 30 sec, 80 °C for 25 sec) followed by 4 °C for 15 min; **e**, 95 °C for 10 min, 9 x (95 °C for 15 sec, 68-60 °C for 1 min each, 81,5 °C for 30 sec) followed by 35 x (95 °C for 15 sec, 60 °C for 1 min, 81,5 °C for 30 sec, 86 °C for 3 sec) followed by 4 °C for 15 min; **f**, 94 °C for 4 min, 35 x (94 °C for 30 sec, 56 °C for 40 sec, 72 °C for 2 min, 80 °C for 25 sec) followed by 4 °C for 15 min. Amplification parameters for q-PCR: **g**, 94 °C for 10 min, 50 x (95 °C for 5 sec, 53 °C for 5 sec, 72 °C for 15 sec, 74 °C for 3 sec) followed by 95 °C for 1 min, 45 °C for 1 min, 65 °C for 1 sec and 40 °C for 10 sec; **h**, 95 °C for 10 min, 50 x (95 °C for 5 sec, 58 °C for 15 sec, 72 °C for 25 sec, 78 °C for 3 sec) followed by 95 °C for 1 min, 45 °C for 1 min, 65 °C for 1 sec and 45 °C for 1 min; **i**, 95 °C for 10 min, 50 x (95 °C for 5 sec, 59 °C for 5 sec, 72 °C for 15 sec, 80 °C for 3 sec) followed by 95 °C for 1 min, 45 °C for 1 min, 65 °C for 1 sec and 40 °C for 10 sec; **j**, 95 °C for 10 min, 50 x (95 °C for 5 sec, 68 °C for 10 sec, 82 °C for 25 sec, 86 °C for 3 sec) followed by 95 °C for 1 min, 45 °C for 1 min, 65 °C for 1 sec and 40 °C for 10 sec; ***k***, 95 °C for 10 min, 50 x (95 °C for 5 sec, 50 °C for 10 sec, 72 °C for 20 sec, 78 °C for 3 sec) followed by 95 °C for 1 min, 45 °C for 1 min, 65 °C for 1 sec and 40 °C for 10 sec.

Holmes, D.E., Nevin, K.P., and Lovley, D.R. (2004). Comparison of 16S rRNA, nifD, recA, gyrB, rpoB and fusA genes within the family Geobacteraceae fam. nov. *Int J Syst Evol Micr* 54**,** 1591-1599.

Li, M., Hong, Y., Klotz, M.G., and Gu, J.D. (2010). A comparison of primer sets for detecting 16S rRNA and hydrazine oxidoreductase genes of anaerobic ammonium-oxidizing bacteria in marine sediments. *Appl Microbiol Biotechnol* 86**,** 781-790.

Lund, M.B., Smith, J.M., and Francis, C.A. (2012). Diversity, abundance and expression of nitrite reductase (nirK)-like genes in marine thaumarchaea. *ISME J* 6**,** 1966-1977.

Mincer, T.J., Church, M.J., Taylor, L.T., Preston, C., Kar, D.M., and Delong, E.F. (2007). Quantitative distribution of presumptive archaeal and bacterial nitrifiers in Monterey Bay and the North Pacific Subtropical Gyre. *Environ Microbiol* 9**,** 1162-1175.

Mosier, A.C., and Francis, C.A. (2010). Denitrifier abundance and activity across the San Francisco Bay estuary. *Env Microbiol Rep* 2**,** 667-676.

Sintes, E., Bergauer, K., De Corte, D., Yokokawa, T., and Herndl, G.J. (2013). Archaeal amoA gene diversity points to distinct biogeography of ammonia-oxidizing Crenarchaeota in the ocean. *Environ Microbiol* 15**,** 1647-1658.

Wuchter, C., Abbas, B., Coolen, M.J., Herfort, L., Van Bleijswijk, J., Timmers, P., et al. (2006). Archaeal nitrification in the ocean. *Proc Natl Acad Sci U S A* 103**,** 12317-12322.
