# Supplementary material for: Niche Differentiation of Aerobic and Anaerobic Ammonia Oxidizers in a High Latitude Deep Oxygen Minimum Zone
Source: Front Microbiol. 2019 Sep 13;10:2141. doi: 10.3389/fmicb.2019.02141 (PMC6753893; doi:10.3389/fmicb.2019.02141)
Supplement: Table S3 — Primer sets and cycling conditions used to amplify bacterial, archaeal, and anammox phylogenetic and functional genes for next-generation sequencing. [file Table_3.DOCX]

**Table S3.** Primer sets and cycling conditions used to amplify bacterial, archaeal and anammox phylogenetic and functional genes for next-generation sequencing.

| **Process** | **Target taxon** | **Gene** | **Primer set** | **Sequence** | **Annealing temperature (°C)** | **Fragment length**  **(bp)** | **Reference** |
| --- | --- | --- | --- | --- | --- | --- | --- |
|  | Bacteria | 16S rRNA | Bakt 341 F | 5‘-CCT ACG GGN GGC WGC AG-3‘ | 51 | 464 | (Klindworth et al., 2013) |
|  |  |  | Bakt 805 R | 5‘-GGA CTA CHV GGG TWT CTA AT-3‘ |  |  |  |
|  | Archaea | 16S rRNA | Arch 349 F | 5‘-GYG CAS CAG KCG MGA AW-3‘ | 50 | 457 | (Takai and Horikoshi, 2000) |
|  |  |  | Arch 806 R | 5‘-GGA CTA CCA GGG TAT CTA AT-3‘ |  |  |  |
|  | Anammox | 16S rRNA | Brod 541 F | 5‘-GAG CAC GTA GGT GGG TTT GT-3‘ | 59 | 208 | (Li et al., 2010) |
|  |  |  | Amx 820 R | 5‘-AAA ACC CCT CTA CTT AGT GCC C-3‘ |  |  |  |
| Nitrification | Archaea | *amo*A | Cren amo F | 5’-ATG GTC TGG CTA AGA CGM TGT A-3’ | 55 | 632 | (Hallam et al., 2006) |
|  |  |  | amoA R | 5’-GCG GCC ATC CAT CTG TAT GT-3’ |  |  | (Francis et al., 2005) |
| Denitrification | Bacteria | *nir*K | nirK-q-F | 5‘-TCA TGG TGC TGC CGC GYG A-3‘ | 68-60 | 472 | (Mosier and Francis, 2010) |
|  |  |  | nirK-1040 | 5‘-GCC TCG ATC AGR TTR TGG TT-3‘ |  |  |  |
|  | Archaea | *nir*K-a | anirK-a 58F | 5‘-ACB YTA TTC GGA AGY ACA TAC ACA-3‘ | 50 | 521 | (Lund et al., 2012) |
|  |  |  | anirK-a 579R | 5‘-GYM ATT CCG TAC ATK CCG GA-3‘ |  |  |  |
|  | Archaea | *nir*K-b | anirK-b 61F | 5‘-CTA TTC GGA RGT WCT TTY ACT GC-3‘ | 50 | 494 | (Lund et al., 2012) |
|  |  |  | anirK-b 555R | 5‘-ACG TGT TGG TCC ATT GCT GC-3‘ |  |  |  |

Francis, C.A., Roberts, K.J., Beman, J.M., Santoro, A.E., and Oakley, B.B. (2005). Ubiquity and diversity of ammonia-oxidizing archaea in water columns and sediments of the ocean. *Proc Natl Acad Sci U S A* 102**,** 14683-14688.

Hallam, S.J., Mincer, T.J., Schleper, C., Preston, C.M., Roberts, K., Richardson, P.M., et al. (2006). Pathways of carbon assimilation and ammonia oxidation suggested by environmental genomic analyses of marine Crenarchaeota. *PLoS Biol* 4**,** e95.

Klindworth, A., Pruesse, E., Schweer, T., Peplies, J., Quast, C., Horn, M., et al. (2013). Evaluation of general 16S ribosomal RNA gene PCR primers for classical and next-generation sequencing-based diversity studies. *Nucleic Acids Research* 41.

Li, M., Hong, Y., Klotz, M.G., and Gu, J.D. (2010). A comparison of primer sets for detecting 16S rRNA and hydrazine oxidoreductase genes of anaerobic ammonium-oxidizing bacteria in marine sediments. *Appl Microbiol Biotechnol* 86**,** 781-790.

Lund, M.B., Smith, J.M., and Francis, C.A. (2012). Diversity, abundance and expression of nitrite reductase (nirK)-like genes in marine thaumarchaea. *ISME J* 6**,** 1966-1977.

Mosier, A.C., and Francis, C.A. (2010). Denitrifier abundance and activity across the San Francisco Bay estuary. *Env Microbiol Rep* 2**,** 667-676.

Takai, K., and Horikoshi, K. (2000). Rapid detection and quantification of members of the archaeal community by quantitative PCR using fluorogenic probes. *Appl Environ Microbiol* 66**,** 5066-5072.
