# Supplementary material for: Niche Differentiation of Aerobic and Anaerobic Ammonia Oxidizers in a High Latitude Deep Oxygen Minimum Zone
Source: Front Microbiol. 2019 Sep 13;10:2141. doi: 10.3389/fmicb.2019.02141 (PMC6753893; doi:10.3389/fmicb.2019.02141)
Supplement: Table S4 — Spearman's rank correlation coefficients between the different bacterial and archaeal groups and the environmental parameters measured. Only significant results of the bacterial and archaeal community are shown (−0.5 > rs > 0.5). No significant correlations were found for anammox bacteria. Numbers in bold are p ≤ 0.01. Dashes indicate no significant correlation for the specific parameter. AOU, Apparent oxygen utilization; Pot. Temp., potential temperature. [file Table_4.DOCX]

**Table S4.** Spearman’s rank correlation coefficients between the different bacterial and archaeal groups and the environmental parameters measured. Only significant results of the bacterial and archaeal community are shown (-0.5 > rs > 0.5). No significant correlations were found for anammox bacteria. Numbers in bold are p ≤ 0.01. Dashes indicate no significant correlation for the specific parameter. Abbreviations: AOU, Apparent oxygen utilization; Pot. Temp., potential temperature.

|  |  | **Depth** | **Pot. Temp.** | **Salinity** | **AOU** | **Nitrite** | **Nitrate** | **Ammonia** |
| --- | --- | --- | --- | --- | --- | --- | --- | --- |
| Bacteria | Other Bacteria | - | - | - | 0.700 | - | -0.714 | - |
|  | Actinomarinales | **-0.847** | **-0.946** | **-0.912** | - | - | -0.714 | - |
|  | Microtrichales | - | - | - | **0.750** | -0.810 | **-0.952** | - |
|  | Flavobacteriales | **-0.949** | **-0.900** | **-0.900** | - | -0.714 | - | 0.733 |
|  | SAR202 clade | **-0.949** | **0.883** | **0.883** | - | - | - | -0.750 |
|  | Synechococcales | **-0.949** | **-0.900** | **-0.900** | - | - | - |  |
|  | Marinimicrobia | **-0.896** | 0.783 | 0.783 | - | - | - | -0.750 |
|  | Nitrospinales | - | - | - | - | - | - | - |
|  | Phycisphaerales | 0.685 | - | - | - | - | - | - |
|  | Other Alphaproteobacteria | - | - | -0.776 | -0.783 | 0.714 | 0.714 | - |
|  | Parvibaculales | -0.738 | 0.767 | **-0.817** | -0.683 | - | - | 0.683 |
|  | Puniceispirillales | - | - | -0.720 | - | - | - | - |
|  | Rhodobacterales | -0.685 | - | -0.700 | -0.783 | - | 0.714 | - |
|  | Rhodospirillales | - | **-0.900** | - | - | - | - | - |
|  | SAR11 clade | 0.738 | - | **0.867** | - | - | - | - |
|  | SAR324 clade | - | **-0.817** | **-** | **0.950** | **-0.857** | **0.976** | -0.717 |
|  | Alteromonadales | **0.843** | - | **0.867** | - | -0.786 | - | **-0.817** |
|  | Betaproteobacterales | - | - | - | - | - | - | - |
|  | Cellvibrionales | **-0.896** | **0.817** | **-0.833** | - | - | - | - |
|  | Chromatiales | - | - | - | - | - | - | - |
|  | Other Gammaproteobacteria | - | - | - | - | - | - | - |
|  | HOC36 | **0.953** | **-0.946** | **0.979** | - | - | - | -0.686 |
|  | Oceanospirillales | - | -0.667 | - | - | - | - | - |
|  | SAR86 clade | - | - | -0.887 | **-0.800** | - | -0.738 | - |
|  | Thiomicrospirales | - | - | 0.683 | 0.750 | - | 0.738 | - |
|  | Thiotrichales | **-0.843** | -0.700 | -0.767 | - | - | - | - |
|  | UBA10353 | 0.685 | - | - | 0.733 | **-0.857** | - | **-0.933** |
|  | Other Proteobacteria | - | - | - | - | - | - | - |
|  | Verrumicrobiales | - | - | - | **-0.850** | 0.762 | -0.762 | - |
| Archaea | Archaea unclassified | - | - | - | - | - | - | - |
|  | Euryarchaeota unclassified | 0.743 | **-0.853** | **0.809** | - | - | - | - |
|  | Halobacteria (Halomicrobiaceae) | 0.794 | -0.778 | **0.845** | - | -0.731 | - | **-0.803** |
|  | Marine Group II | **-0.843** | **0.900** | **-0.917** | - | - | - | 0.667 |
|  | Marine Group III | **0.949** | **-0.917** | **0.900** | - | - | - | - |
|  | Thermoplasmata unclassified | - | - | - | - | - | - | - |
|  | Nanoarchaeota unclassified | - | - | - | - | -0.733 | - | - |
|  | Woesearchaeia | **0.953** | **-0.912** | **0.879** | - | - | - | -0.728 |
|  | Marine Benthic Group A | **0.965** | **-0.932** | **0.915** | - | -0.708 | - | -0.763 |
|  | Nitrosopumilaceae | **0.685** | **-0.750** | **0.800** | - | - | - | - |
|  | Nitrososphaeria unclassified | - | - | - | - | - | - | - |
|  | Thaumarchaeota unclassified | 0.750 | - | - | - | -0.708 | -0.781 | - |
