# Supplementary material for: Niche Differentiation of Aerobic and Anaerobic Ammonia Oxidizers in a High Latitude Deep Oxygen Minimum Zone
Source: Front Microbiol. 2019 Sep 13;10:2141. doi: 10.3389/fmicb.2019.02141 (PMC6753893; doi:10.3389/fmicb.2019.02141)
Supplement: Table S5 — Diversity parameters for phylogenetic (based on 16S rRNA) and functional prokaryotic groups. nitrif., nitrification; amoA, archaeal amoA genes; nirK-a and nirK-b, archaeal nitrite reductase K variants a and b; bac nirK, bacterial nitrite reductase K; epi, epipelagic zone; OMZ, oxygen minimum zone; bathy, bathypelagic zone; nseqs, number of sequences sampled for OTU definition; inv. Simpson, inverse of the Simpson index; sobs, number of OTUs observed. Numbers in bold are significantly different (Kruskal–Wallis ANOVA on rank, p < 0.004). [file Table_5.DOCX]

**Table S5.** Diversity parameters for phylogenetic (based on 16S rRNA) and functional prokaryotic groups. Abbreviations: nitrif., nitrification; *amo*A, archaeal *amo*A genes; *nir*K-a and *nir*K-b, archaeal nitrite reductase K variants a and b; bac *nir*K, bacterial nitrite reductase K; epi, epipelagic zone; OMZ, oxygen minimum zone; bathy, bathypelagic zone; nseqs, number of sequences sampled for OTU definition; inv. Simpson, inverse of the Simpson index; sobs, number of OTUs observed. Numbers in bold are significantly different (Kruskal-Wallis ANOVA on rank, p < 0.004).

|  |  |  | **nseqs** | **Chao1** | **Sobs** | **Shannon** | **Inv. Simpson** |
| --- | --- | --- | --- | --- | --- | --- | --- |
| 16S rRNA | Bacteria | Epi | 3451 - 3784 | 6627 - 11073 | 1727 - 2230 | 6.35 – 7.11 | 75.78 – 307.36 |
|  |  | OMZ | 3121 - 4163 | 8285 - 11340 | 1901 - 2367 | 6.64 - 6.77 | 86.10 – 98.15 |
|  |  | Bathy | 2655 - 3418 | 6479 - 9349 | 1316 - 1961 | 5.98 - 6.62 | 63.82 – 109.57 |
|  | Archaea | Epi | 5461 - 7110 | **418 - 429** | **196 - 199** | **1.43 – 1.64** | 2.43 – 2.73 |
|  |  | OMZ | 4755 - 7400 | 968 - 1561 | **421 - 534** | **3.05 - 3.19** | 7.52 – 8.38 |
|  |  | Bathy | 4927 - 8656 | **1029 - 1392** | **446 - 512** | **2.62 - 3.25** | 4.93 – 7.57 |
|  | Anammox | Epi | 11720 - 14769 | 9798 - 10295 | 4336 - 4932 | 6.99 - 7.00 | 64.14 - 65.23 |
|  |  | OMZ | 3286 - 9794 | 6477 - 7661 | 1760 - 3271 | 6.52 - 6.59 | 45.64 - 80.40 |
|  |  | Bathy | 8959 | 9788 | 3777 | 7.00 | 78.51 |
| DENITRIFICATION | *nir*K-a | Epi | 665 - 5566 | 145 - 4209 | 37 - 1375 | 1.59 - 5.21 | 2.47 - 32.97 |
|  |  | OMZ | 389 - 5245 | 3437 - 9936 | 319 - 2268 | 5.10 - 6.47 | 10.82 - 252.39 |
|  |  | Bathy | 1060 - 5387 | 2194 - 6549 | 537 - 1676 | 5.22 - 5.40 | 23.84 - 53.96 |
|  | *nir*K-b | Epi | 1765 - 5776 | **600 - 2330** | **166 - 660** | **2.33 - 3.34** | **4.65 - 6.40** |
|  |  | OMZ | 6580 - 8737 | **5402 - 6490** | **1712 - 2122** | **4.38 - 4.84** | 7.53 - 9.92 |
|  |  | Bathy | 2050 - 8250 | 1442 - 4387 | 486 - 1327 | 4.03 - 4.39 | **9.61 - 12.74** |
|  | bac *nir*K | Epi | 5211 | 4361 | 681 | 2.36 | 3.29 |
|  |  | Bathy | 3569 - 5882 | 6417 - 12023 | 659 - 1423 | 2.21 - 5.66 | 3.22 - 26.29 |
| NITRIF. | *amo*A | Epi | 6215 - 9761 | 12778 - 20057 | **1685 - 2756** | 4.00 - 4.41 | 5.54 - 7.50 |
|  |  | OMZ | 1385 - 1705 | 4146 - 8919 | 629 - 919 | 4.94 - 6.03 | 28.13 - 99.35 |
|  |  | Bathy | 811 - 1065 | 2048 - 4434 | **349 - 538** | 4.32 - 5.55 | 9.87 - 46.05 |
