# Supplementary figures and images for: Niche Differentiation of Aerobic and Anaerobic Ammonia Oxidizers in a High Latitude Deep Oxygen Minimum Zone
Source: Front Microbiol. 2019 Sep 13;10:2141. doi: 10.3389/fmicb.2019.02141 (PMC6753893; doi:10.3389/fmicb.2019.02141)

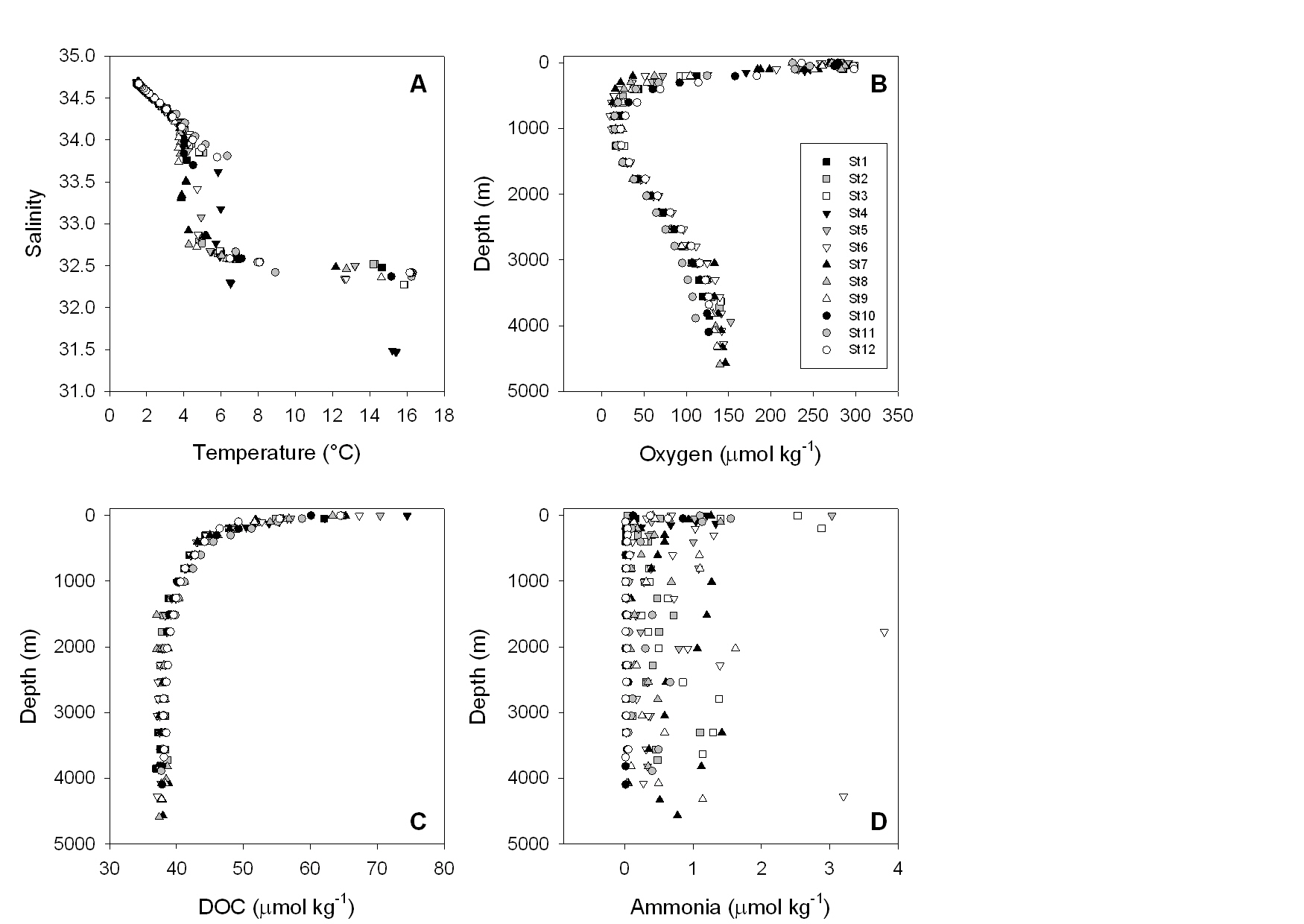

Supplement: Figure S1 — Salinity-temperature diagram of the different stations sampled in the Gulf of Alaska (A). Depth profiles of dissolved oxygen (B), dissolved organic carbon (C), and ammonia (D) concentration in different stations. [file Image_1.JPEG]

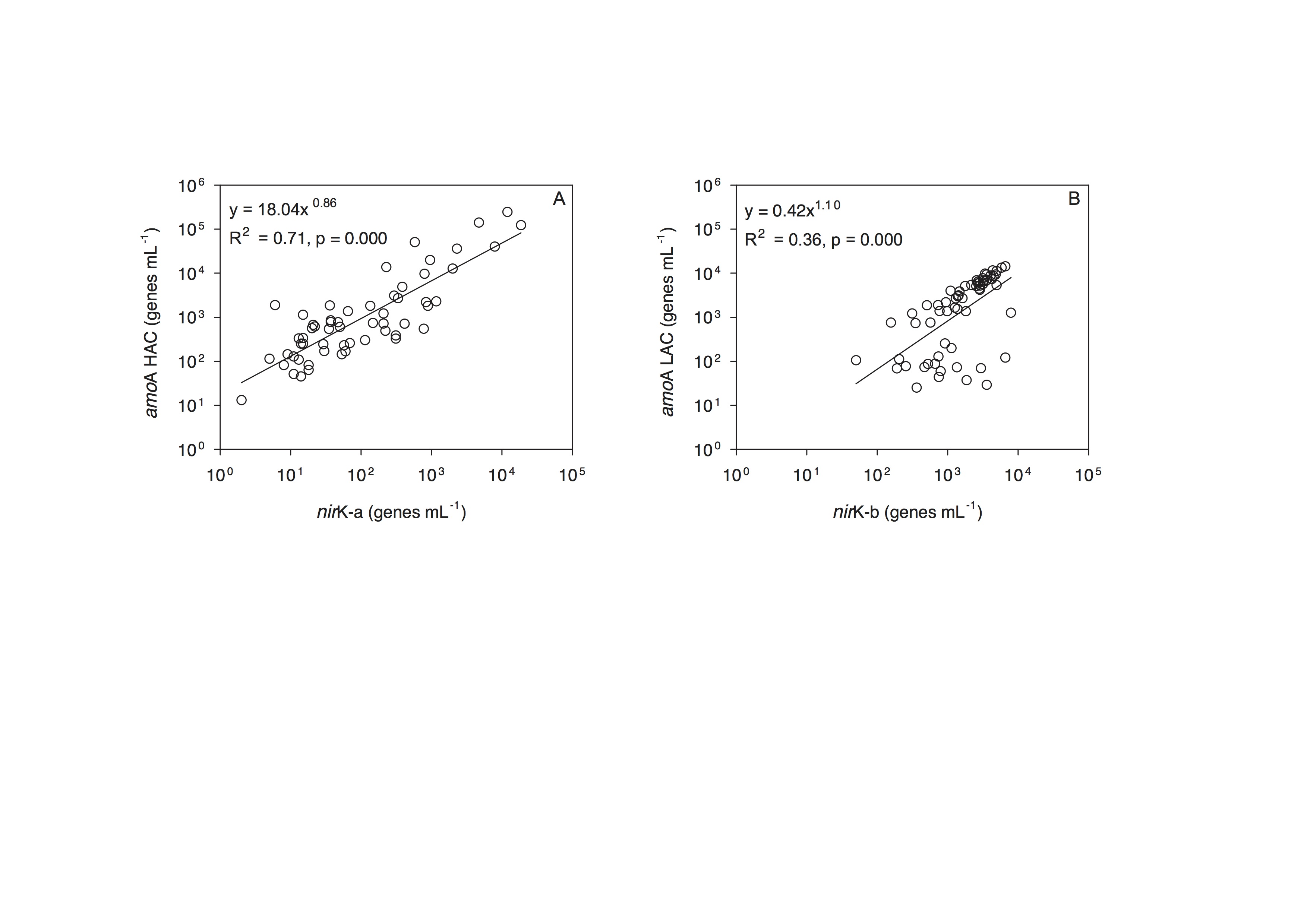

Supplement: Figure S2 — Correlation between archaeal denitrification (nirK-a, nirK-b) and nitrification (amoA HAC, amoA LAC) genes. (A) “High-ammonia concentration” archaeal amoA (amoA HAC) vs. archaeal nitrate reductase K variant a (nirK-a); (B) “low-ammonia concentration” archaeal amoA (amoA LAC) vs. archaeal nitrate reductase K variant b (nirK-b). [file Image_2.JPEG]

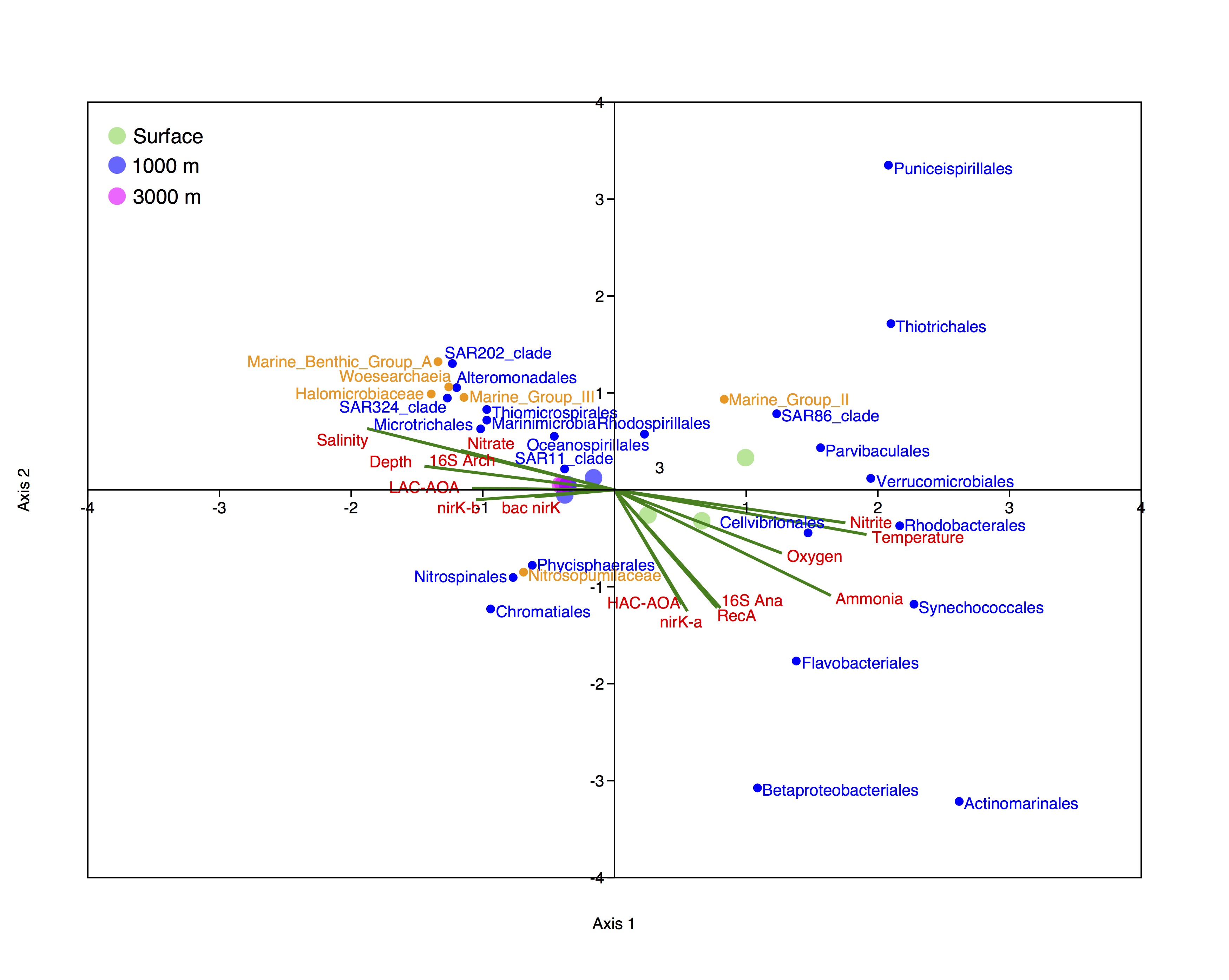

Supplement: Figure S3 — Canonical correspondence analysis of the bacterial and archaeal phylotypes using the environmental parameters and the phylogenetic and functional gene abundance. [file Image_3.JPEG]

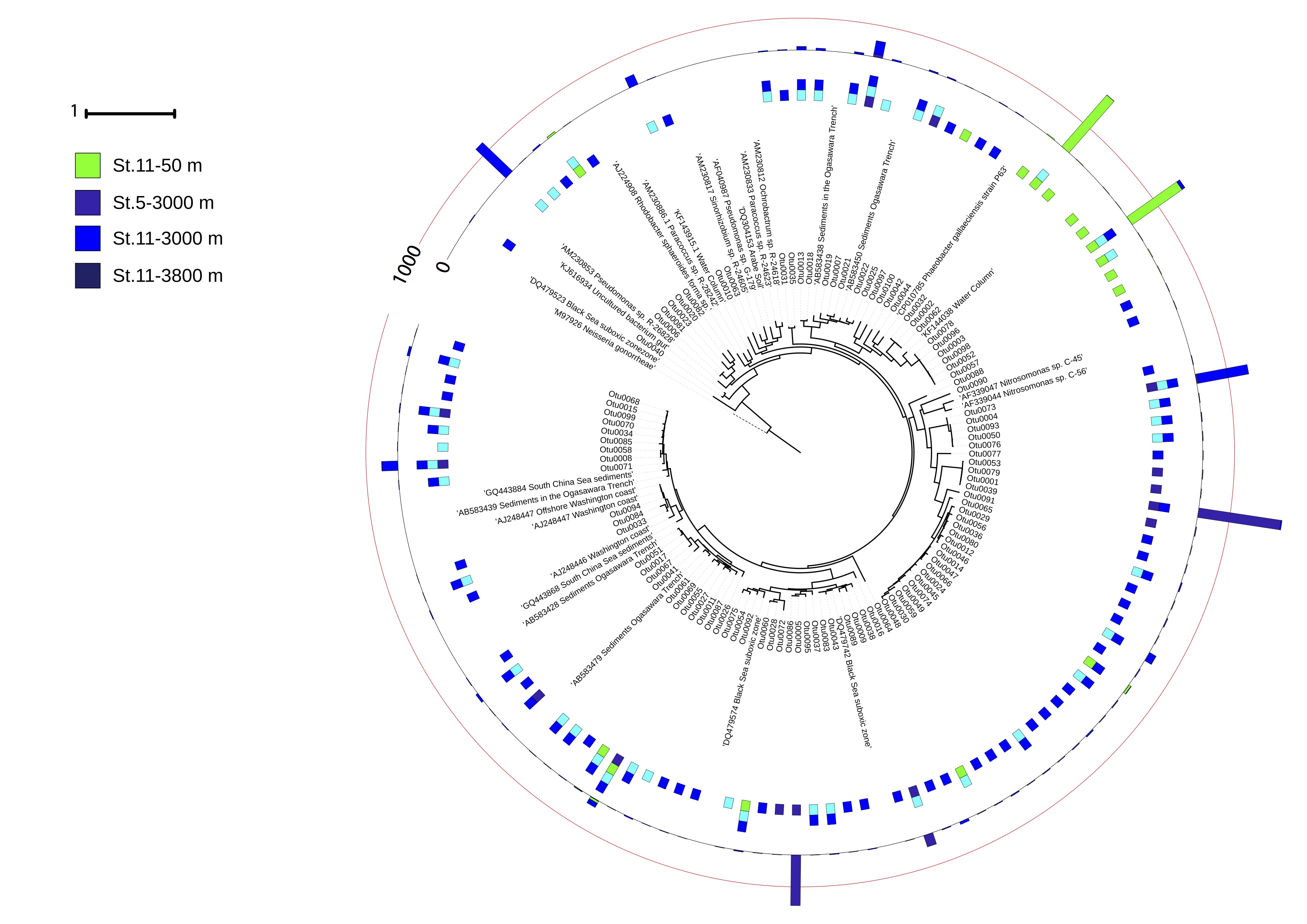

Supplement: Figure S4 — Phylogenetic tree of the 100 most abundant bacterial nirK OTUs sampled in the Gulf of Alaska. One representative of each sequence group >97% identical is shown; the bar shows the number of sequences represented by the OTU, and the color indicates the depth layer. Green tones: epipelagic; dark blue tones: bathypelagic. Reference and environmental sequences are included. [file Image_4.JPEG]
